# ﻿A new, widespread genus of Baetidae from South Asia (Insecta, Ephemeroptera)

**DOI:** 10.3897/zookeys.1168.104844

**Published:** 2023-07-03

**Authors:** Thomas Kaltenbach, Nikita J. Kluge, Jean-Luc Gattolliat

**Affiliations:** 1 Muséum cantonal des Sciences Naturelles, Département de zoologie, Palais de Rumine, Place Riponne 6, CH-1005 Lausanne, Switzerland Muséum cantonal des Sciences Naturelles, Département de zoologie, Palais de Rumine Lausanne Switzerland; 2 University of Lausanne (UNIL), Department of Ecology and Evolution, CH-1015 Lausanne, Switzerland University of Lausanne (UNIL) Lausanne Switzerland; 3 Department of Entomology, Biological Faculty, Saint-Petersburg State University, Universitetskaya nab., 7/9, Saint Petersburg, 199034, Russia Saint Petersburg State University Saint Petersburg Russia

**Keywords:** Brunei, Indonesia, mayflies, Sri Lanka, Taiwan

## Abstract

Material collected on different islands across South Asia revealed a new genus of Baetidae with a widespread distribution, *Arcobaetis***gen. nov.** The larvae present important similarities with *Nigrobaetis*, but have paraglossae dorsally with an arc of long, spine-like setae in distal area; long, slightly feathered setae between prostheca and mola of both mandibles; and very slender legs with row of short setae at dorsal margin of femur. The male imago has an extraordinarily small 3^rd^ (apical) segment of gonostylus, which is much narrower than the apex of the 2^nd^ segment. The new genus includes five species: *A.sumbawensis***sp. nov.** is described from Sumbawa (Indonesia) based on larvae, *A.sumatrensis***sp. nov.** from Sumatra (Indonesia) based on larvae, *A.bornensis***sp. nov.** from Borneo (Brunei) based on larvae, and *A.sripadai***sp. nov.** (type species) is described from Sri Lanka based on a reared male imago with its larval and subimaginal exuviae; *A.gracilentus* (Chang & Yang, 1994), **comb. nov.** from Taiwan, formerly described in Margobaetis Kang & Yang, 1994, a subgenus of Baetis Leach, 1815, and subsequently transferred to the genus *Nigrobaetis* Kazlauskas (in Novikova & Kluge), 1987, is transferred to the new genus. A key to the larvae of all species is provided. Morphological similarities and the relationship of the new genus to other genera of Baetidae are discussed.

## ﻿Introduction

Baetidae are a highly diverse, cosmopolitan family of mayflies, missing only in New Zealand from among places with mayflies (Gattolliat and Nieto 2009; Kluge 2023). They comprise nearly one third of all mayfly species and approximately one quarter of all mayfly genera worldwide (Jacobus et al. 2019; Kluge 2023). Actually, there are ca. 115 valid genera of Baetidae worldwide, and approximately 34 of them are found in South Asia, depending on whether these clades are considered as genera or subgenera. Collection and research activities in this region were strongly increasing during the last two decades. Consequently, several new genera and subgenera of Baetidae were recently described, and other genera were recor­ded the first time (Kaltenbach et al. 2020b, 2021, 2022, 2023; Kluge 2020a, 2020b, 2022; Kluge and Suttinun 2020; Suttinun et al. 2020; Phlai-ngam et al. 2022a).

Here, we describe and illustrate a new genus of Baetidae, *Arcobaetis* gen. nov., with a wide distribution across South Asia, including the islands Sumbawa, Sumatra, Borneo, Sri Lanka, and Taiwan. It includes one known species from Taiwan, *A.gracilentus* comb nov., formerly described in Margobaetis Kang & Yang, 1994, a subgenus of Baetis, and subsequently transferred to the genus *Nigrobaetis* and now transferred to *Arcobaetis* gen. nov., and four new species, which are described and illustrated based on larvae (*A.sumbawensis* sp. nov. from Sumbawa, *A.sumatrensis* sp. nov. from Sumatra, and *A.bornensis* from Borneo), and in one case based on a male imago together with its larval and subimaginal exuviae (*A.sripadai* sp. nov. from Sri Lanka).

The new genus is distinguished from all other genera of Baetidae by the following combination of larval characters: frons with carina-like elevation; both mandibles with long setae between prostheca and mola; paraglossae dorsally with an arc of long, spine-like setae in distal area; very slender legs with femora length 4–6× maximum width; femora with row of short, spine-like setae at dorsal margin; claws with a single row of denticles, distal denticles larger and directed distad, proximal denticles minute; subimaginal gonostyli under cuticle of male last instar larva folded in the “*Nigrobaetis*-type”. Male imago with an extraordinary small 3^rd^ (apical) segment of gonostylus, much narrower than apex of 2^nd^ segment.

Considering the generally extreme species diversity in South Asia, the rather poor collection activities in the past with the exception of the last two decades, with many still unexplored regions, and the obvious richness of Baetidae in this region, and examination and re-evaluation of historical collections in light of new interpretations, we have to expect further new genera and many more species in the future.

## ﻿Materials and methods

The larvae were collected by kick-sampling and preserved in 70%-96% ethanol. The specimens from Brunei were collected in 2014 by Kate Baker (University of Exeter, UK) during ecological studies in Brunei Darussalam in collaboration with Universiti Brunei Darussalam (Baker et al. 2016a, b).

A subimago was reared by one of us (NK) from a mature larva in a glass with stagnant water. Subsequently, the male imago was reared from the subimago in a container with wet air, but without water. The imago was associated with its larval and subimaginal exuviae.

The dissection of larvae was done in Cellosolve (2-Ethoxyethanol) with subsequent mounting on slides with Euparal liquid, using an Olympus SZX7 stereomicroscope. Alternatively, dissection was done in alcohol with subsequent mounting on slides with Canada balsam, using a stereomicroscope MSP 2; and examination with microscope Leica DM 1000.

The DNA of two specimens of one species (*A.sumbawensis* sp. nov.) was extracted using non-destructive methods allowing subsequent morphological analysis (see Vuataz et al. 2011 for details). We amplified a 658 bp fragment of the mitochondrial gene cytochrome oxidase subunit 1 (COI) using the primers LCO 1490 and HCO 2198 (Folmer et al. 1994). Sequencing was done with Sanger’s method (Sanger et al. 1977). The genetic variability between two specimens was estimated using Kimura-2-parameter distances (K2P, Kimura 1980), calculated with the program MEGA 11 (Tamura et al. 2021, http://www.megasoftware.net).

Drawings were made using an Olympus BX43 microscope. To facilitate the determination of species and the comparison of important structures, we partly used a combination of dorsal and ventral aspects in one drawing. Explanations are given in Kaltenbach et al. (2020a: fig. 1).

**Figure 1.**
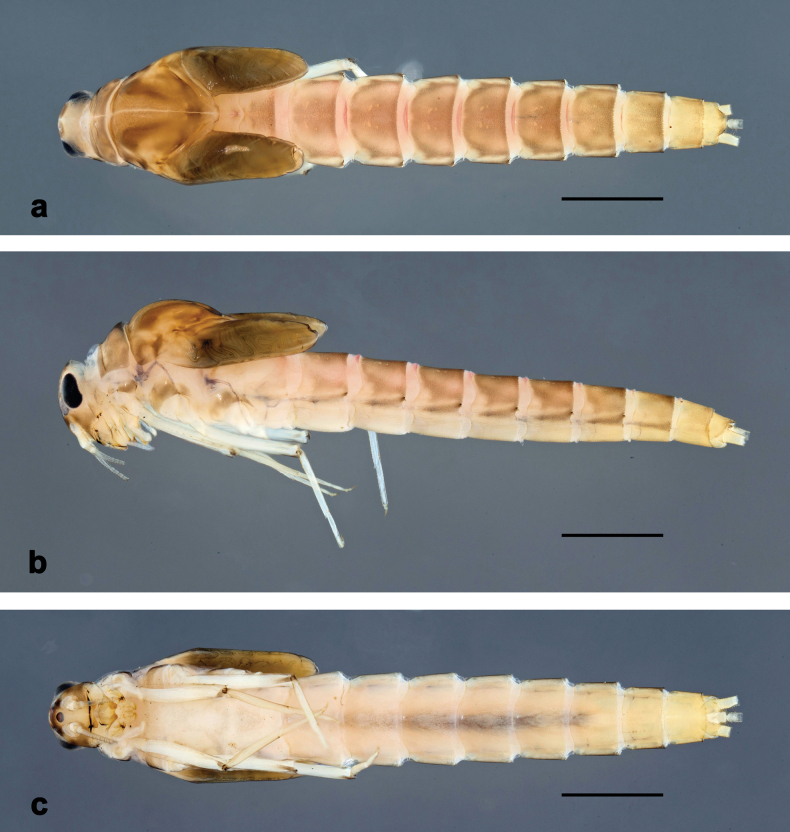
*Arcobaetissumbawensis* sp. nov., larva, habitus (holotype) **a** dorsal view **b** lateral view **c** ventral view. Scale bars: 1 mm

Photographs of larvae in toto were taken using a Canon EOS 6D camera and processed with the programs Adobe Photoshop Lightroom (http://www.adobe.com) and Helicon Focus v. 5.3 (http://www.heliconsoft.com). Images of larval, subimaginal, and imaginal parts were taken with a DMC 4500 camera on a Leica M205C stereomicroscope, and an Olympus SC 50 camera on an Olympus BX43 microscope, processed with the program Olympus Cell Sense v. 3.2. SEM pictures were taken using a FEI Quanta FEC 250 electron microscope (Thermo Fisher). Photographs were subsequently enhanced with Adobe Photoshop Elements 13.

The distribution maps were generated with the program SimpleMappr (https://simplemappr.net, Shorthouse 2010), GPS coordinates of sample locations are given in Table 2. Google Earth (http://www.google.com/earth/download/ge/) was used to attribute approximate GPS coordinates to sample locations from Taiwan, based on Kang et al. (1994).

The dichotomous key was elaborated with the support of the program DKey v. 1.3.0 (http://drawwing.org/dkey, Tofilski 2018).

The terminology follows Kluge (2004).

### ﻿Abbreviations

**MZB**Museum Zoologicum Bogoriense (Indonesia)

**MZL**Muséum cantonal des Sciences Naturelles, Lausanne (Switzerland)

**SPbU** Saint-Petersburg State University (Russia)

## ﻿Results

### ﻿Taxonomic account

#### ﻿ Arcobaetis gen. nov.

Taxon classificationAnimaliaEphemeropteraBaetidae

BA0315B0-1ED0-5A1D-B3B0-2229F76A78EE

https://zoobank.org/F4E4945E-62ED-4E71-80A0-B993DA84EB57

Figs 1 , 2 , 3 , 4 , 5 , 6 , 7 , 8 , 9 , 10 , 11 , 12 , 13 , 14 , 15 , 16 , 17 , 18 , 19

##### Type species.

*Arcobaetissripadai* sp. nov., by present designation.

Species included in *Arcobaetis* gen. nov.

1. *Arcobaetissumbawensis* sp. nov.

2. *Arcobaetissumatrensis* sp. nov.

3. *Arcobaetisbornensis* sp. nov.

4. *Arcobaetissripadai* sp. nov.

5. *Arcobaetisgracilentus* (Chang & Yang, in Kang et al. 1994), comb. nov.

##### Diagnosis.

**Figure 2.**
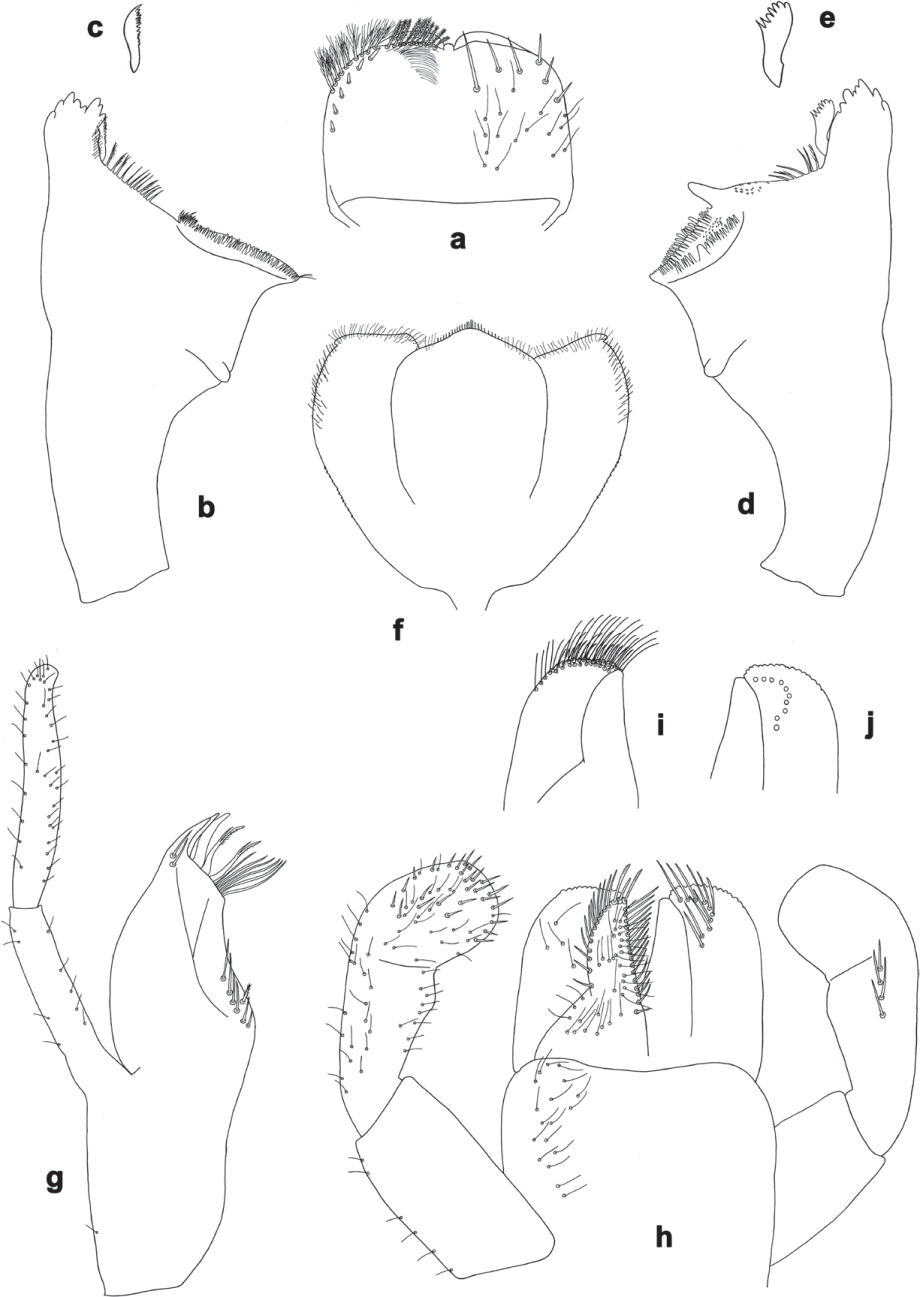
*Arcobaetissumbawensis* sp. nov., larva **a** labrum (left: ventral view; right: dorsal view) **b** right mandible **c** right prostheca **d** left mandible **e** left prostheca **f** hypopharynx and superlinguae **g** maxilla **h** labium (left: ventral view; right: dorsal view) **i** apex of paraglossa (ventral view) **j** apex of paraglossa (dorsal view).

**Figure 3.**
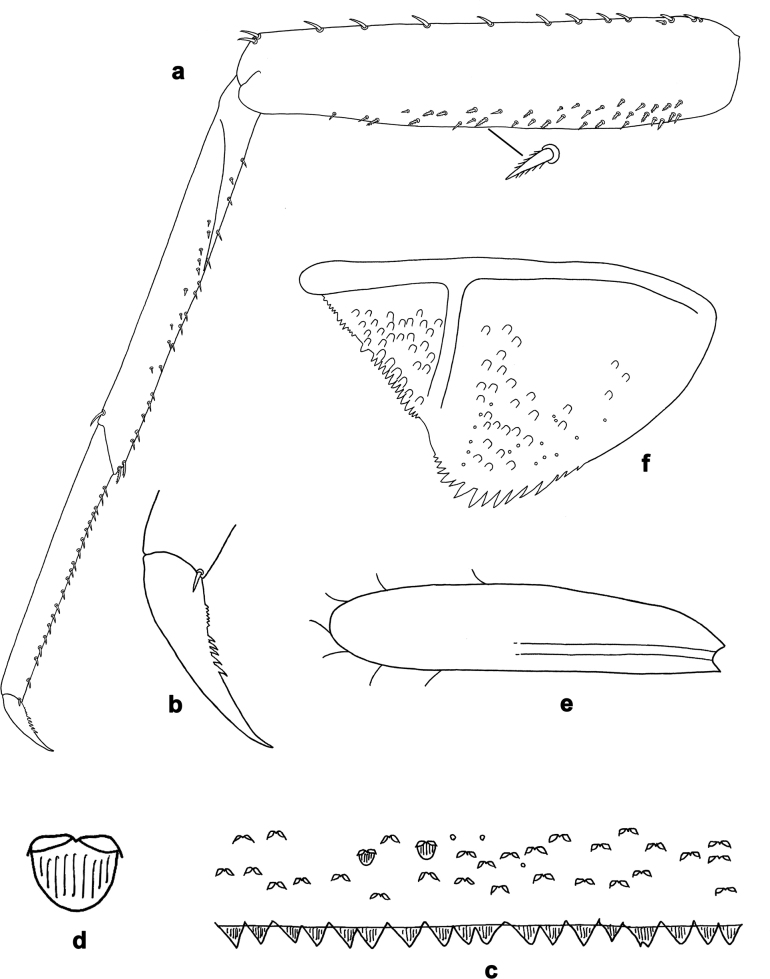
*Arcobaetissumbawensis* sp. nov., larva **a** foreleg **b** fore claw **c** tergum IV **d** scale on tergum IV **e** tergalius I **f** paraproct.

**Figure 4.**
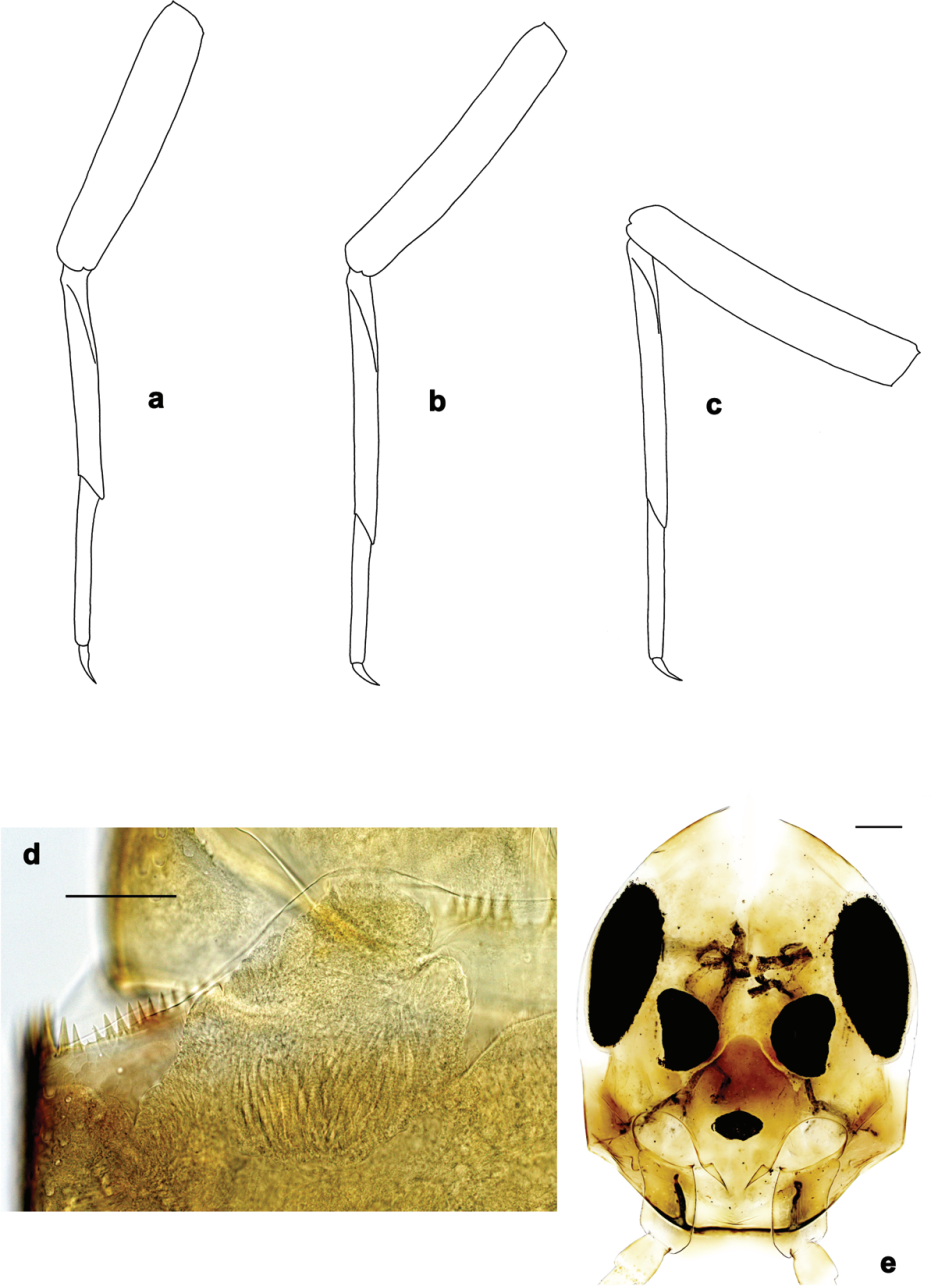
*Arcobaetissumbawensis* sp. nov., larva **a** foreleg **b** middle leg **c** hind leg **d** subimaginal gonostylus developing under cuticle of last instar male larva **e** head. Scale bars: 0.05 mm (**e**); 0.1 mm (**d**).

**Figure 5.**
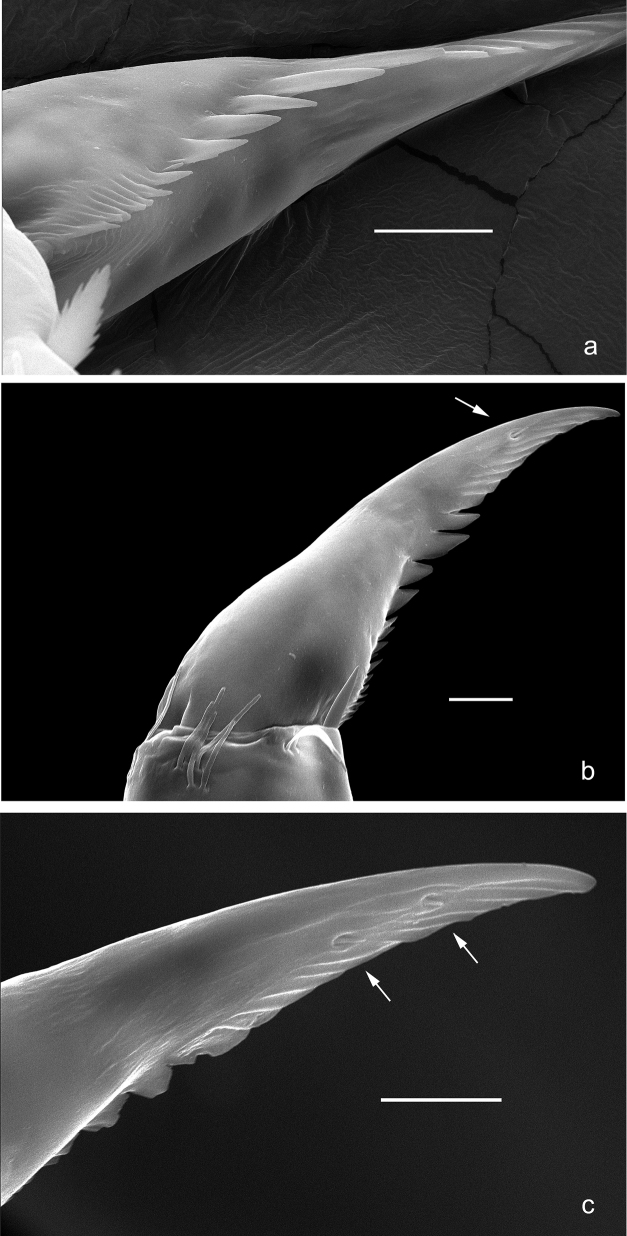
*Arcobaetissumbawensis* sp. nov., larva, SEM pictures **a** fore claw, lateroventral view **b** middle claw, anterior view **c** middle claw, posterior view. Arrows: subapical setae. Scale bars: 0.01 mm.

**Larva.** The following combination of characters differentiate *Arcobaetis* gen. nov. from all other genera of Baetidae: A) frons with carina-like elevation, slightly overlapping antennal base (Fig. 4e); B) maxillary palp with two segments (Fig. 2g); C) both mandibles with outermost denticle in ventral position, set apart from other denticles; both mandibles with long, slightly feathered setae between prostheca and mola (Fig. 7b, d); D) paraglossae dorsally with an arc of long, spine-like, simple setae in distal area (Fig. 2h, j); E) labial palp segment II with poorly developed distomedial protuberance (Fig. 2h); F) legs very slender; femur length 4–6× maximum width; outer margin of femora with row of short, spine-like setae (Fig. 4a–c); G) claw with single row of denticles, distal denticles larger and directed distad, proximal denticles minute (Figs 3b, 5a, b); H) surface of abdominal terga with fine, longitudinally striated scales situated in angulate nests, whose angles bear opercula (Fig. 3c, d); H) folding of subimaginal gonostyli developing under cuticle of last instar male larva of the “*Nigrobaetis*-type”.

##### Description.

**Larva. *Head*** (Fig. 4e). Frons with carina-like elevation, slightly overlapping antennal base.

***Labrum*** (Fig. 2a). Sub-rectangular, wider than long. Distal margin with medial, shallow emargination and small process. Dorsally with pair of long, simple, sub-median setae, and on each side partial sub-marginal arc of long, stout, simple setae; sometimes with setae between sub-median seta and partial sub-marginal arc; surface scattered with medium to long, simple setae. Ventrally with long long-feathered setae on anterolateral margin and medium short-feathered setae on medial margin; several small, stout setae near anterolateral and sometimes also midlateral margin.

***Right mandible*** (Fig. 2b, c). Incisor and kinetodontium fused; incisor with denticles, outer denticle turned ventrally and much lower than other denticles; kinetodontium with denticles; inner margin of innermost denticle of kinetodontium with row of thin setae; prostheca stick-like, distolaterally denticulate; margin between prostheca and mola straight, with long, slightly feathered setae; apex of mola with tuft of setae.

***Left mandible*** (Fig. 2d, e). Incisor and kinetodontium fused; incisor with denticles, outer denticle turned ventrally and set apart from other denticles; kinetodontium with denticles; prostheca robust, distally denticulate; margin between prostheca and mola straight, with row of long, slightly feathered setae; apex of mola without tuft of setae.

Both mandibles with outer lateral margins almost straight.

***Hypopharynx and superlinguae*** (Fig. 2f). Apex of lingua with poorly developed tuft of setae. Distolateral margin of superlinguae with fine setae.

***Maxilla*** (Fig. 2g). Apically with three slender, pointed canines and three denti-setae; distal denti-seta tooth-like, bent in the same direction as canines; other denti-setae slender, bifid, and pectinate; maxillary palp with two segments, apex rounded.

***Labium*** (Fig. 2h). Glossae basally broad, narrowing towards apex, shorter than paraglossae; inner margin with row of spine-like setae, increasing in length distally; apex with several medium to long, robust setae; outer margin with row of spine-like setae; ventral surface with numerous medium to long fine, simple scattered setae. Paraglossae laterally straight, distally slightly bent inwards; apex with three rows of long, robust, distally pectinate setae; ventrally usually with several short, simple setae in distomedial area; dorsally with distal arc of long, spine-like setae. Labial palp with three segments, segment II with slightly developed distomedial protuberance.

***Thorax*. *Legs*** (Figs 3a, b, 4a–c). Long and very slender. ***Femur.*** Length approx. 4–6× maximum width; outer margin with row of curved, spine-like setae; apex rounded, with pair of curved, spine-like setae; ventrally with stout, pointed, pectinate setae along margin; femoral patch absent. ***Tibia.*** Outer margin of fore legs almost bare, with one curved, spine-like seta at apex; middle and hind legs additionally with row of few spine-like setae. ***Tarsus.*** Dorsal margin almost bare. ***Claw.*** Slender, with long point, and with one row of denticles. Distal denticles larger and directed distad, proximal denticles are minute.

**Abdomen. *Terga*** (Fig. 3c, d). Surface with irregular rows of scale bases and with fine, longitudinally striated scales situated in angulate nests, whose angles bear opercula (see also Kluge 2012: 365, fig. 6).

***Tergalii.*** Present on segments I–VII or II–VII.

***Paraproct*** (Fig. 3f). Posterior margin with stout spines. Cercotractor with numerous, small, marginal spines.

***Larval protogonostyli*** (Fig. 4d). Subimaginal gonostyli developing under cuticle of last instar male larvae folded in “*Nigrobaetis*-type”: all segments (1^st^, 2^nd^, and 3^rd^) directed caudally and compressed in longitudinal direction, base of 2^nd^ segment deeply inserted into 1^st^ segment, and 3^rd^ segment inserted into 2^nd^ segment (as in Novikova and Kluge 1994: fig. 2: 1, 5, 20).

##### Diagnoses.

**Imago.** Following combination of characters differentiate *Arcobaetis* gen. nov. from other genera of Baetidae A) forewing with double intercalary veins (Fig. 17a); B) gonostylus segment III extraordinary small, much narrower than apex of 2^nd^ segment (Fig. 18a); C) gonovectes sharply bent (Fig. 18a).

##### Description.

**Male Imago.** See description of male imago under *A.sripadai* sp. nov. below.

##### Etymology.

*Arcobaetis* is a combination of *Arco*-, in reference to the Latin word arcus for arc and the arc of long, simple setae dorsodistally on paraglossae, and *baetis*, to highlight the similarities with the genus *Baetis*. The gender is masculine.

##### Distribution

**(Fig. 19).** Indonesia (Sumatra, Sumbawa), Brunei, Sri Lanka, Taiwan.

#### ﻿ Arcobaetis sumbawensis sp. nov.

Taxon classificationAnimaliaEphemeropteraBaetidae

6E4FA03D-5D32-522D-A54F-BD02C733CE43

https://zoobank.org/7A575C67-8D0D-4A93-BB2B-BF7669B229D5

Figs 1 , 2 , 3 , 4 , 5 , 19

##### Type material.

***Holotype*.** Indonesia • male larva (last instar); Sumbawa, Batu Dulang, Mt. Batu Pasak, forest streams; 08°37'42"S, 117°15'27"E, SUMB09; 1380 m; 17.ix.2011; leg. M. Balke; on slide; GBIFCH00975680; MZB. ***Paratypes*.** 9 larvae; same data as holotype; 4 on slides; GBIFCH00692615, GBIFCH00975688, GBIFCH00592652, GBIFCH00592653; 5 in alcohol; GenBank OQ699910, OQ699911; GBIFCH00975695, GBIFCH00975689, GBIFCH00975690, GBIFCH00975694; MZL.

##### Diagnosis.

**Larva** (Table 1). The following combination of characters distinguish *A.sumbawensis* sp. nov. from other species of *Arcobaetis* gen. nov.: A) distal margins of segments in middle part of flagellum without enlarged spines; B) labial palp segment III sub-rectangular, at base approx. as wide as distal margin of segment II (Fig. 2h); C) hind protoptera absent; D) claw with single row of denticles, four or five distalmost denticles larger, ca. ten basal denticles small to minute (Figs 3b, 5a); E) tergalii present on abdominal segments I–VII; F) posterior margin of tergum IV with triangular spines, slightly wider than long (Fig. 3c).

**Table 1.** States of selected larval characters of *Arcobaetis* gen. nov. Figure numbers refer to those in this paper, while those of *A.gracilentus* comb. nov. refer to Kang et al. (1994).

Characters	*A.sumbawensis* sp. nov.	Fig.	*A.sumatrensis* sp. nov.	Fig.	*A.bornensis* sp. nov.	Fig.	*A.sripadai* sp. nov.	Fig.	*A.gracilentus* comb. nov.	Fig.
	Sumbawa		Sumatra		Borneo		Sri Lanka		Taiwan	
Flagellum, enlarged spines on distal margins of segments	absent		absent		present (middle part)	11f	absent	14a	absent	
Labial palp segment III	sub-rectangular, base approx. as wide as distal margin of segment II	2h	sub-rectangular, base narrower than distal margin of segment II	7h	sub-quadrangular, base narrower than distal margin of segment II	10h	sub-quadrangular, base approx. as wide as distal margin of segment II	14i	sub-quadrangular, base narrower than distal margin of segment II	7E
Hind protoptera	absent		absent		absent		absent		present	7G
Tergalii on abdominal segments	I–VII		II–VII		I–VII		II–VII		I–VII	
Abdominal tergite IV, spines on posterior margin	triangular	3c	triangular	8i	triangular	11c	triangular	15f	triangular	7K
	slightly wider than long		wider than long		wider than long		slightly wider than long		longer than wide	
Cerci, spines on outer sides	unknown		two somewhat longer on each 2^nd^ segment	9f	unknown		several strongly enlarged on each 2^nd^ segment	15o, p	unknown	

##### Description.

**Larva** (Figs 1–5). Body length 5.7–7.0 mm.

***Cuticular colouration*** (Fig. 1a–c). Head, thorax, and abdomen dorsally brown, thorax darker. Head, thorax, and abdomen ventrally ochre. Legs ochre, femur distally with brown streak; tibia basally along patella-tibial suture darker. Caudalii ochre.

***Hypodermal colouration.*** Abdomen dorsally with narrow reddish transverse stripes on intersegmental membranes (Fig. 1a).

***Antenna.*** Flagellum in middle part without enlarged spines at distal margin of segments.

***Labrum*** (Fig. 2a). Length 0.7× maximum width. Dorsally with sub-median seta, short sub-marginal arc of simple setae, and two setae in between.

***Right mandible*** (Fig. 2b, c). Incisor with five denticles; kinetodontium with three denticles.

***Left mandible*** (Fig. 2d, e). Incisor with five denticles; kinetodontium with three denticles.

***Hypopharynx and superlinguae*** (Fig. 2f). Lingua as long as superlinguae, longer than broad. Superlinguae distally straight; lateral margins rounded; fine, long, simple setae along distal margin.

***Maxilla*** (Fig. 2g). Galea-lacinia ventrally with two simple setae just proximad of canines. Medially with one pectinate, spine-like seta and ca. six short to long, simple setae. Maxillary palp approx. 1.5× as long as galea-lacinia; palp segment II approx. 1.3× as long as segment I; setae on maxillary palp fine, simple, scattered over surface of segments I and II.

***Labium*** (Fig. 2h–j). Inner margin of glossa with ca. 16 spine-like setae, increasing in length distally; apex with two long and one medium robust setae; outer margin with ca. 12 spine-like setae; paraglossa ventrally with ca. four medium, simple setae in anteromedial area; dorsally with arc of ca. 10 long, spine-like setae in distal area. Labial palp with segment I 0.7× length of segments II and III combined. Segment II dorsally with row of ca. four spine-like setae. Segment III sub-rectangular; at base approx. as wide as distal margin of segment II; ventral surface with short, spine-like, simple setae and short, fine, simple setae.

***Hind protoptera*** absent.

***Legs*** (Figs 3a, b, 4a–c, 5a–c). Long and slender, middle, and hind legs slenderer than fore legs. Ratio of leg segments: fore leg 1.3:1.0:0.7:0.2, middle and hind legs 1.2:1.0:0.6:0.1. ***Femur***. Length of fore femur ca. 5× maximum width, outer and inner margins almost parallel; length of middle and hind femora >6× maximum width, outer margins slightly concave. Outer margin with row of ca. 12 short, curved, spine-like setae, on fore leg larger than on middle and hind legs. On ventral side with short, spine-like, pectinate, pointed setae; larger and denser on foreleg, smaller and less dense on middle and hind legs. ***Tibia***. Short, stout, pointed, pectinate setae near inner margin. Inner margin with row of short, curved, spine-like, pectinate setae, on apex two longer, curved, spine-like, pectinate setae. Outer margin with one apical, spine-like seta; on fore leg without other spine-like setae, on middle and hind legs with row of few medium, spine-like setae. Patella-tibial suture on proximal ¹/3 of tibia on all legs. ***Tarsus***. Inner margin with row of short, curved, pectinate, spine-like setae; outer margin bare. ***Claw*** with one row of denticles; four or five distalmost denticles larger and directed distad; basally ca. 10 small to minute denticles; two vestigial subapical setae on anterior side, one vestigial subapical seta on posterior side (Fig. 5a, b).

***Abdominal terga*** (Fig. 3c, d). Posterior margin of terga: I smooth, without spines; II–IX with triangular spines, increasing in length toward IX; slightly wider than long on tergum IV; row of spines on tergum IX interrupted by smaller spines in middle part behind bases of submedian setae (similar to Fig. 15j). Posterior margin of tergum X with median concavity with smaller spines (similar to Fig. 15k).

***Abdominal sterna.*** Posterior margins of sterna: I–V smooth, without spines; VI–IX with triangular spines. On sternum IX of male mature larva row of narrow pointed spines between protogonostyli, smaller spines laterad of protogono­styli and larger, pointed spines laterad of them (similar to Fig. 15m).

***Tergalii*** (Fig. 3e). Present on abdominal segments I–VII. Tracheation in tergalius I limited to basal part of main trunk. In nine well preserved larvae, only a few tergalii were found, but insertions were always well developed: two tergalii I in one specimen; one tergalius IV and one tergalius V in another specimen, both very small, looking like replacements of naturally broken tergalii.

***Paraproct*** (Fig. 3f). Posterior margin with ca. 16 stout spines. Surface scattered with scale bases and micropores.

***Caudalii*.** Cerci and paracercus with broad triangular spines on posterior margin of each segment.

##### Etymology.

The specific epithet refers to the type locality in Sumbawa (Indonesia).

##### Distribution.

Indonesia: Sumbawa (Fig. 19).

##### Biological aspects.

The species was found at an altitude of 1380 m.

#### ﻿ Arcobaetis sumatrensis sp. nov.

Taxon classificationAnimaliaEphemeropteraBaetidae

2DBE6903-B10D-5ECD-9C35-2A6A07DD03A4

https://zoobank.org/D595E315-4847-481C-9297-1792ADF392D4

**Figure 6.**
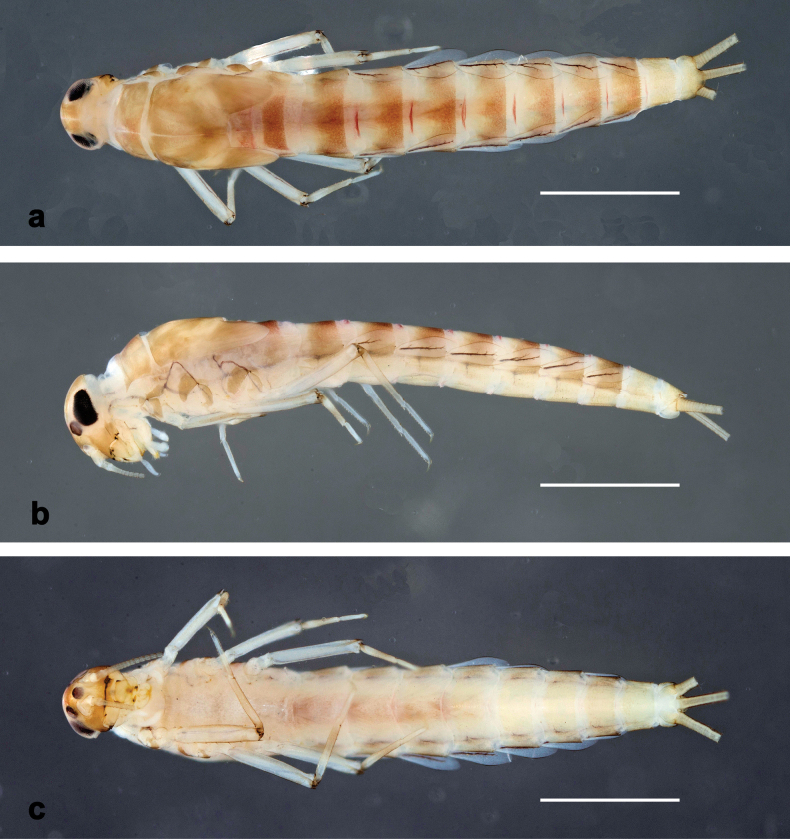
*Arcobaetissumatrensis* sp. nov., larva, habitus (holotype) **a** dorsal view **b** lateral view **c** ventral view. Scale bar: 1 mm

**Figure 7.**
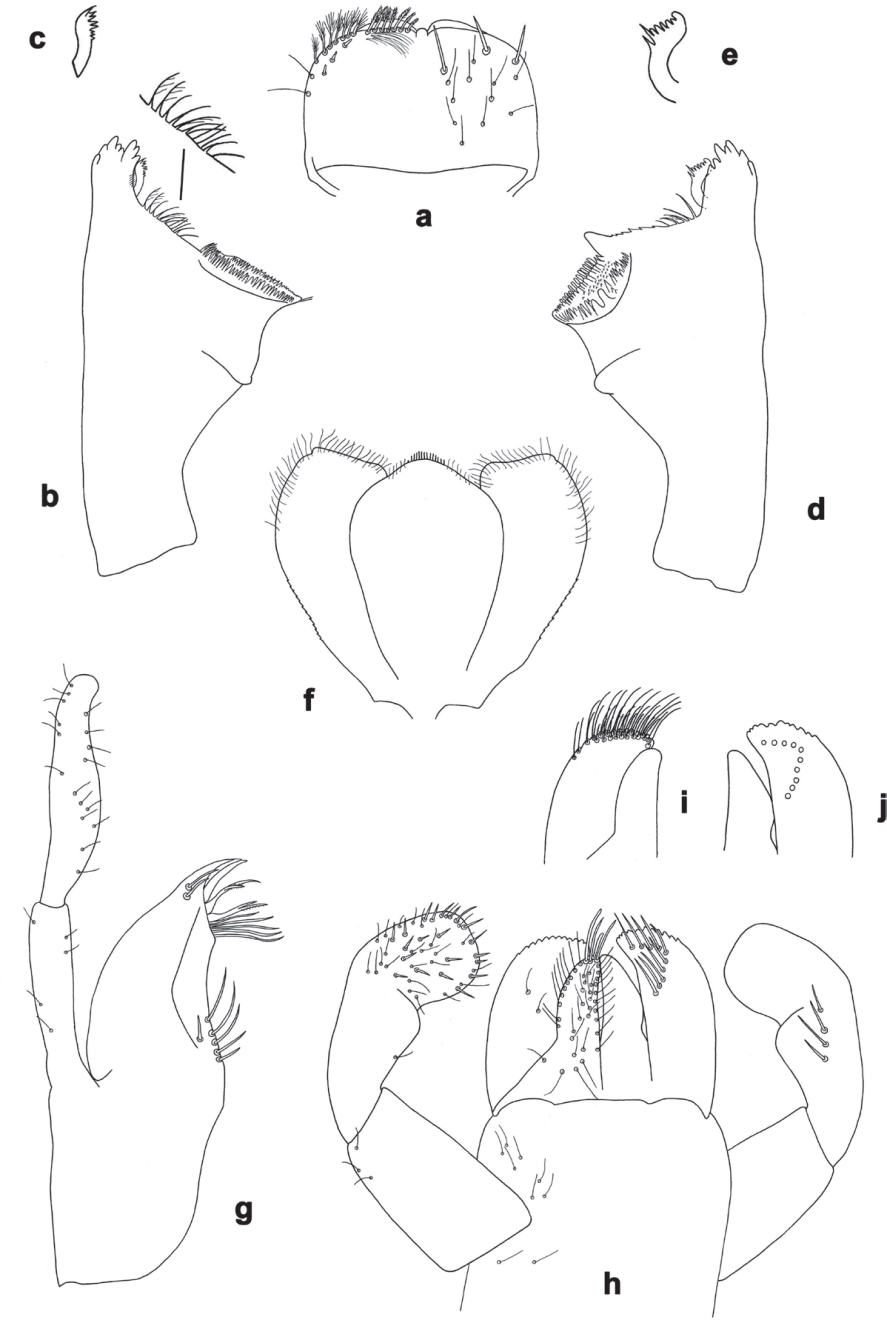
*Arcobaetissumatrensis* sp. nov., larva **a** labrum (left: ventral view; right: dorsal view) **b** right mandible **c** right prostheca **d** left mandible **e** left prostheca **f** hypopharynx and superlinguae **g** maxilla **h** labium (left: ventral view; right: dorsal view) **i** apex of paraglossa (ventral view) **j** apex of paraglossa (dorsal view).

**Figure 8.**
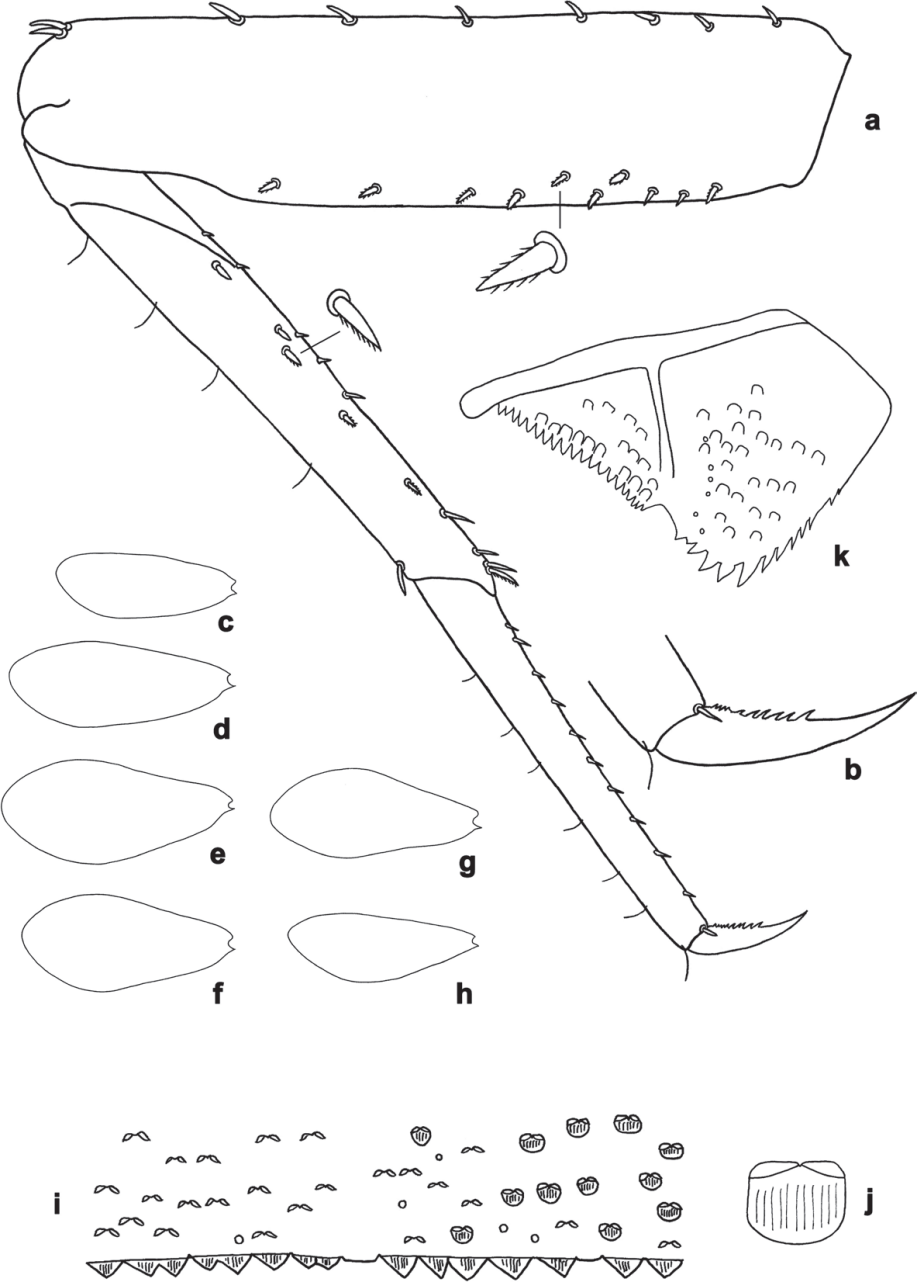
*Arcobaetissumatrensis* sp. nov., larva **a** foreleg **b** fore claw **c** tergalius II **d** tergalius III **e** tergalius IV **f** tergalius V **g** tergalius VI **h** tergalius VII **i** tergum IV **j** scale on tergum IV **k** paraproct.

**Figure 9.**
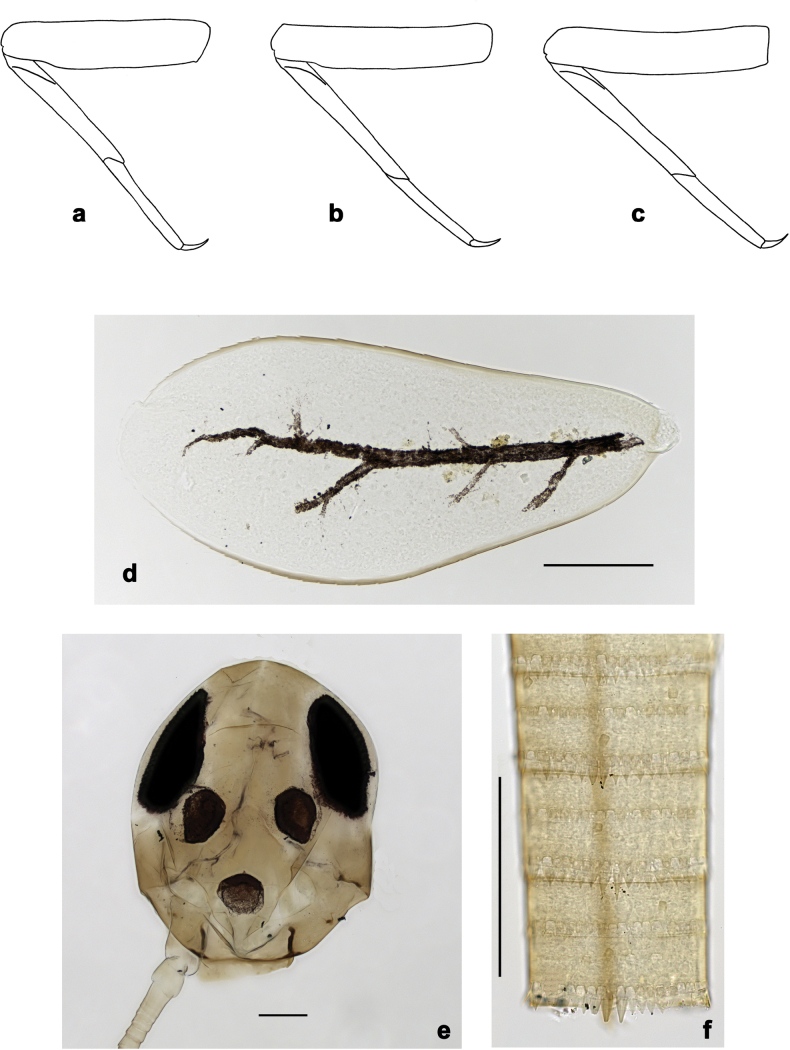
*Arcobaetissumatrensis* sp. nov., larva **a** foreleg **b** middle leg **c** hind leg **d** tergalius IV **e** head **f** fragment of cercus (lateral view). Scale bars: 0.1 mm.

Figs 6 , 7 , 8 , 9 , 19

##### Type material.

***Holotype*.** Indonesia • female larva (premature); Sumatra Barat, Harau Canyon, stream near Ikbal’s cottage, UN11; 00°06'26"S, 100°40'22"E; 520 m; 23.vi.2012; leg. M. Balke; on slide; GBIFCH00592617; MZB. ***Paratypes*.** 1 larva; same data as holotype; on slide; GBIFCH00975679; MZL • 1 larva; Sumatra Barat, Bukit Barisan, above Padang, creek, UN3; 00°56'44"S, 100°32'44"E; 1047 m; 8.xi.2011; leg. M. Balke; on slide; GBIFCH00975704; MZL.

##### Diagnosis.

**Larva** (Table 1). The following combination of characters distinguish *A.sumatrensis* sp. nov. from other species of *Arcobaetis* gen. nov.: A) distal margins of segments in middle part of flagellum without enlarged spines; B) labial palp segment III sub-rectangular, at base smaller than distal margin of segment II (Fig. 7h); C) hind protoptera absent; D) claw with single row of denticles, ca. six distalmost denticles larger, other denticles minute (Fig. 8b); E) tergalii present on abdominal segments II–VII (Fig. 8c–h; F) posterior margin of tergum IV with triangular spines, wider than long (Fig. 7i).

##### Description.

**Larva** (Figs 6–9). Body length ca. 4.6 mm.

***Cuticular colouration*** (Fig. 6a–c). Head, thorax, and abdomen dorsally brown, ventrally ochre. Legs ochre, joining of femur and tibia dark brown; middle part of tibia pale brown. Caudalii pale brown.

***Hypodermal colouration.*** Abdomen dorsally with narrow reddish transverse stripes on intersegmental membranes (Fig. 6a).

***Antenna*.** Flagellum in middle part without enlarged spines at distal margin of segments.

***Labrum*** (Fig. 7a). Length 0.7× maximum width. Dorsally with sub-median seta and sub-marginal arc of two simple setae.

***Right mandible*** (Fig. 7b, c). Incisor with five denticles; kinetodontium with three denticles.

***Left mandible*** (Fig. 7d, e). Incisor with four denticles; kinetodontium with three denticles.

***Hypopharynx and superlinguae*** (Fig. 7f). Lingua as long as superlinguae, longer than broad. Superlinguae distally straight; lateral margins rounded; fine, long, simple setae along distal margin.

***Maxilla*** (Fig. 7g). Galea-lacinia ventrally with two simple setae just proximad of canines. Medially with one pectinate, spine-like seta and ca. five short to long, simple setae. Maxillary palp approx. 1.5× as long as galea-lacinia; palp segment II approx. 1.2× as long as segment I; setae on maxillary palp fine, simple, scattered over surface of segments I and II.

***Labium*** (Fig. 7h–j). Inner margin of glossa with ca. ten spine-like setae; apex with three long and one medium robust setae; outer margin with ca. nine spine-like setae; paraglossa ventrally with ca. two medium, simple setae in anteromedial area; dorsally with arc of ca. ten long, spine-like setae in distal area. Labial palp with segment I 0.8× length of segments II and III combined. Segment II dorsally with row of ca. four spine-like setae. Segment III sub-rectangular; at base narrower than distal margin of segment II; ventral surface with short, spine-like, simple setae and short, fine, simple setae.

***Hind protoptera*** absent.

***Legs*** (Figs 8a, b, 9a–c). Long and slender, middle, and hind legs slenderer than fore leg. Ratio of leg segments: fore leg 1.4:1.0:0.8:0.2, middle legs 1.3:1.0:0.6:0.2 and hind legs 1.2:1.0:0.6:0.1. ***Femur***. Length of fore femur ca. 4.4× maximum width, outer and inner margins almost parallel; length of middle and hind femora > 5× maximum width, outer margins slightly concave. Outer margin with row of ca. seven short, curved, spine-like setae, on fore leg larger than on middle and hind legs. On ventral side of fore leg with short, spine-like, pectinate, pointed setae; nearly absent on middle and hind legs. ***Tibia***. Short, stout, pointed, pectinate setae irregularly near inner margin. Inner margin with row of short, curved, spine-like, pectinate setae, on apex two longer, curved, spine-like, pectinate setae. Outer margin with one apical, spine-like seta; on fore leg without other spine-like setae, on middle and hind legs with few medium, spine-like setae. Patella-tibial suture on proximal ¹/4 of tibia on all legs. ***Tarsus***. Inner margin with row of short, curved, pectinate, spine-like setae; outer margin without spine-like setae. ***Claw*** with single row of denticles, ca. six distalmost denticles larger and directed distad, other denticles minute.

***Abdominal terga*** (Fig. 8i, j). Posterior margin of terga: I smooth, without spines; II–IV with short triangular spines, wider than long; V with triangular spines approx. as wide as long; VI–IX with triangular spines longer than wide, sharply pointed; row of spines on tergum IX interrupted by smaller spines in middle part behind bases of submedian setae (similar to Fig. 15j).

***Abdominal sterna*.** Posterior margins of sterna: I–V smooth, without spines; VI–IX with triangular spines.

***Tergalii*** (Figs 8c–h, 9d). Present on abdominal segments II–VII. Tracheation partially extending toward inner and outer margins. Margins with minute denticles intercalating short, simple setae.

***Paraproct*** (Fig. 8k). Posterior margin with ca. 13 stout spines. Surface scattered with scale bases and micropores.

***Caudalii*** (Fig. 9f). Cerci and paracercus with small, rather wide, triangular, pointed spines on posterior margin of each segment; on outer side of cerci two somewhat longer spines on each 2^nd^ segment, no such longer spines on paracercus.

##### Etymology.

The specific epithet refers to the type locality in Sumatra (Indonesia).

##### Distribution.

Indonesia: Sumatra (Fig. 12b).

##### Biological aspects.

The species was found at altitudes of 520 m and 1050 m.

#### ﻿ Arcobaetis bornensis sp. nov.

Taxon classificationAnimaliaEphemeropteraBaetidae

267D6366-A4C1-5276-86B8-B3FE40CBBDDD

https://zoobank.org/1FD12A10-3730-4CED-AC63-2EFCE4894444

**Figure 10.**
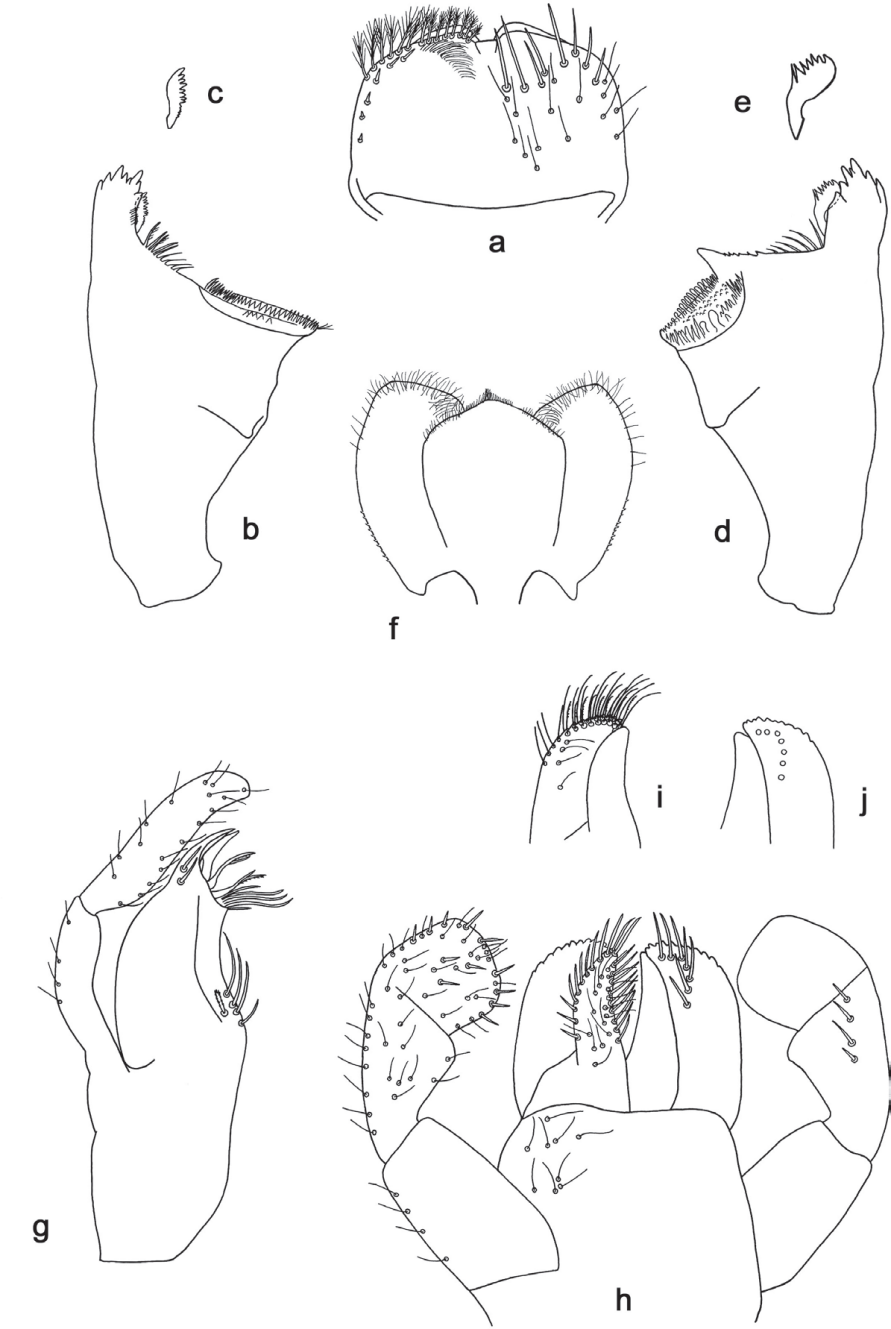
*Arcobaetisbornensis* sp. nov., larva **a** labrum (left: ventral view; right: dorsal view) **b** right mandible **c** right prostheca **d** left mandible **e** left prostheca **f** hypopharynx and superlinguae **g** maxilla **h l**abium (left: ventral view; right: dorsal view) **i** apex of paraglossa (ventral view) **j** apex of paraglossa (dorsal view).

**Figure 11.**
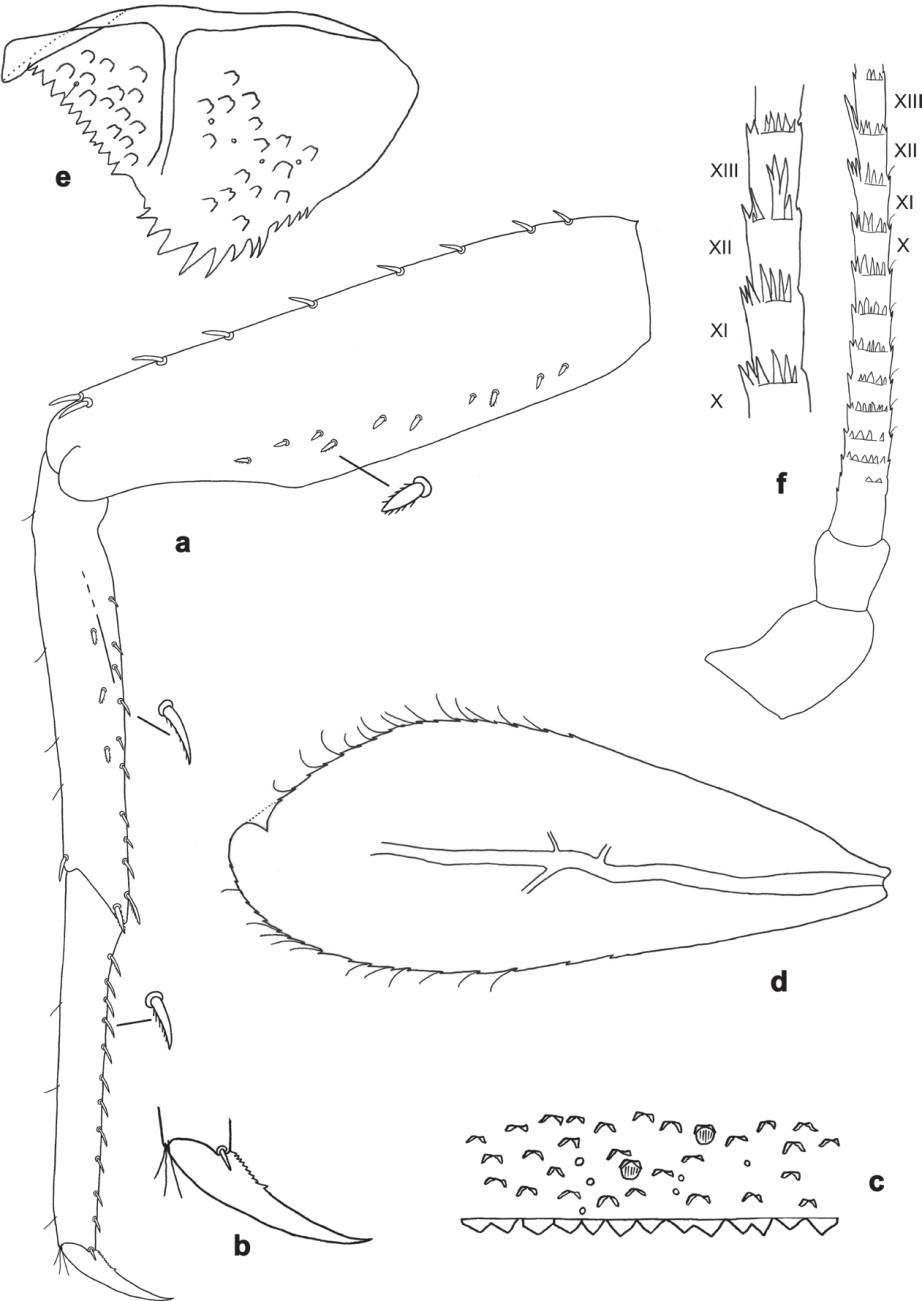
*Arcobaetisbornensis* sp. nov., larva **a** foreleg **b** fore claw **c** tergum IV **d** tergalius IV **e** paraproct **f** antenna (right: dorsal view; left: section in lateral view).

**Figure 12.**
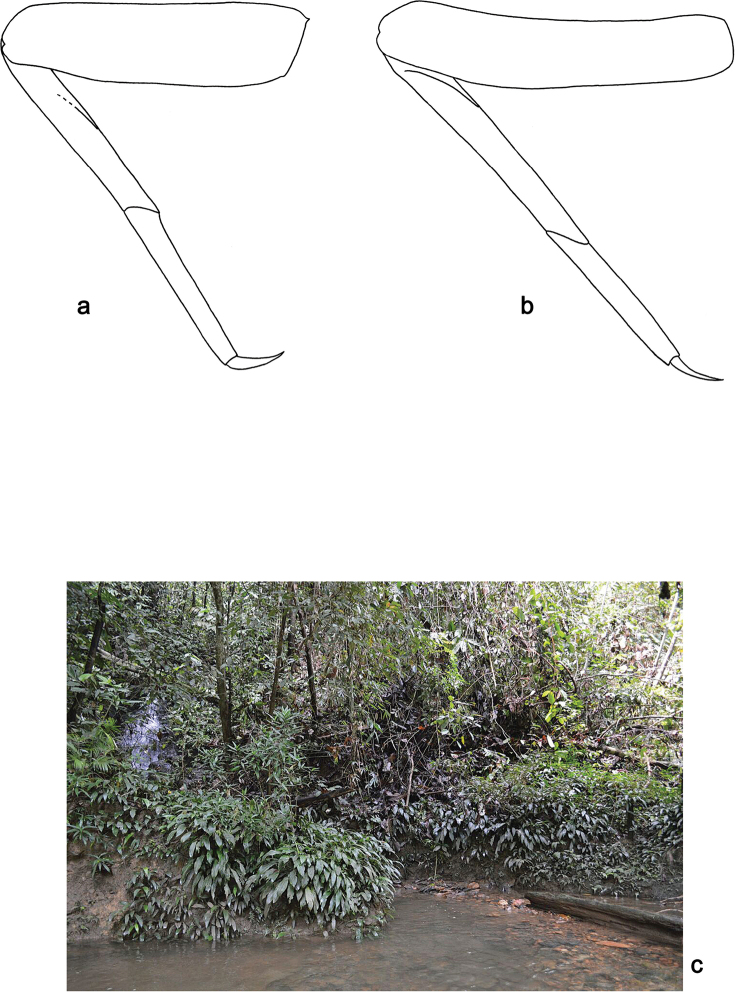
*Arcobaetisbornensis* sp. nov., larva **a** foreleg **b** hind leg **c** habitat (photo Kate Baker, University Exeter, UK).

Figs 10 , 11 , 12 , 19

##### Type material.

***Holotype*.** Brunei • female larva (premature); Temburong District, Ulu Temburong National Park, near Kuala Belalong Field Studies Centre (KBFSC); 04°32'55"N, 115°09'27"E; 103 m; May 2014; leg. Kate Baker; on slide; GBIFCH00465126; MZL. ***Paratypes.*** 11 larvae; same data as holotype; 5 on slides; GBIFCH00465127, GBIFCH00465128, GBIFCH00465129, GBIFCH00592613, GBIFCH00592645; MZL; 6 in alcohol; GBIFCH00515212, GBIFCH00975703; MZL.

##### Diagnosis.

**Larva** (Table 1). The following combination of characters distinguish *A.bornensis* sp. nov. from other species of *Arcobaetis* gen. nov.: A) distal margins of segments in middle part of flagellum with enlarged spines (Fig. 11f); B) labial palp segment III sub-quadrangular, at base narrower than distal margin of segment II (Fig. 10h); C) hind protoptera absent; D) claw with single row of denticles, distalmost denticle larger, other denticles minute (Fig. 11b); E) tergalii present on abdominal segments I–VII; F) posterior margin of tergum IV with triangular spines, wider than long (Fig. 11c).

##### Description.

**Larva** (Figs 10–12). Body length ca. 3.0 mm.

***Colouration***. Head, thorax, and abdomen dorsally brown, ventrally ochre. Legs ecru. Caudalii pale brown.

***Hypodermal colouration.*** Unknown.

***Antenna.*** Flagellum in middle part with enlarged spines at distal margin of segments (Fig. 11f).

***Labrum*** (Fig. 10a). Length 0.7× maximum width. Dorsally with sub-median seta, sub-marginal arc of ca. three simple setae, and several setae in between.

***Right mandible*** (Fig. 10b, c). Incisor with five denticles; kinetodontium with three denticles.

***Left mandible*** (Fig. 10d, e). Incisor with four denticles; kinetodontium with three denticles.

***Hypopharynx and superlinguae*** (Fig. 10f). Lingua slightly shorter than superlinguae, longer than broad. Superlinguae distally straight; lateral margins rounded; fine, long, simple setae along distal margin.

***Maxilla*** (Fig. 10g). Galea-lacinia ventrally with two simple setae just proximad of canines. Medially with one pectinate, spine-like seta and ca. four medium to long, simple setae. Maxillary palp approx. 1.4× as long as galea-lacinia; palp segment II approx. 1.2× as long as segment I; setae on maxillary palp fine, simple, scattered over surface of segments I and II.

***Labium*** (Fig. 10h–j). Inner margin of glossa with ca. nine spine-like setae; apex with three long robust setae; outer margin with ca. nine spine-like setae; paraglossa ventrally with ca. four medium, simple setae in anteromedial area; dorsally with arc of ca. seven long, spine-like setae in distal area. Labial palp with segment I 0.8× length of segments II and III combined. Segment II dorsally with row of ca. four spine-like setae. Segment III sub-quadrangular; at base narrower than distal margin of segment II; ventral surface with short, spine-like, simple setae and short, fine, simple setae.

***Hind protoptera*** absent.

***Legs*** (Figs 11a, b, 12a, b). Long and slender, hind legs slenderer than fore leg, middle legs unknown. Ratio of leg segments: fore leg 1.5:1.0:0.9:0.3, hind leg 1.4:1.0:0.7:0.2. ***Femur*.** Length of fore femur ca. 3.6× maximum width, outer and inner margins almost parallel; length of hind femur ca. 5× maximum width, outer margin slightly concave. Outer margin with row of ca. 7 short, curved, spine-like setae, on fore leg larger than on hind leg. On ventral side of fore leg with short, spine-like, pectinate, pointed setae; nearly absent on hind leg. ***Tibia*.** Few short, stout, pointed, pectinate setae near inner margin. Inner margin with row of short, curved, spine-like, pectinate setae, on apex two longer, curved, spine-like, pectinate setae. Outer margin with one apical, spine-like seta; on fore leg without other spine-like setae, on hind leg with few medium, spine-like setae. Patella-tibial suture on foreleg on proximal ¹/2 (difficult to see), on hind leg on proximal ¹/3. ***Tarsus***. Inner margin with row of short, curved, pectinate, spine-like setae; outer margin without spine-like setae. ***Claw*** with one row of denticles, distalmost denticle larger and directed distad, other denticles minute.

***Abdominal terga*** (Fig. 11c). Posterior margin of terga: I and II smooth, without spines; III–IX with triangular spines, slightly increasing in length toward IX; wider than long on tergum IV; row of spines on tergum IX interrupted by smaller spines in middle part behind bases of submedian setae (similar to Fig. 15j). Posterior margin of tergum X with median concavity with smaller spines (similar to Fig. 15k).

***Abdominal sternites*.** Posterior margins of sterna: I–V smooth, without spines; VI–IX with triangular spines.

***Tergalii*** (Fig. 11d). Present on abdominal segments I–VII. Tracheation limited to main trunk. Margin with minute denticles intercalating short, simple setae.

***Paraproct*** (Fig. 11e). Posterior margin with ca. 13 stout spines. Surface scattered with scale bases and micropores.

***Caudalii*.** Spines of cerci and paracercus unknown.

##### Etymology.

The specific epithet refers to the type locality in Borneo (Brunei).

##### Distribution.

Brunei (Fig. 19).

##### Biological aspects

**(Fig. 12c).** The species was found at an altitude of 1380 m in slow current of a small and shallow stream.

#### ﻿ Arcobaetis sripadai sp. nov.

Taxon classificationAnimaliaEphemeropteraBaetidae

2BD5D6EF-48A5-5C49-8A71-BF8C2D53A131

https://zoobank.org/1883B5F0-BB80-4568-9A6F-F9597991CCE5

**Figure 13.**
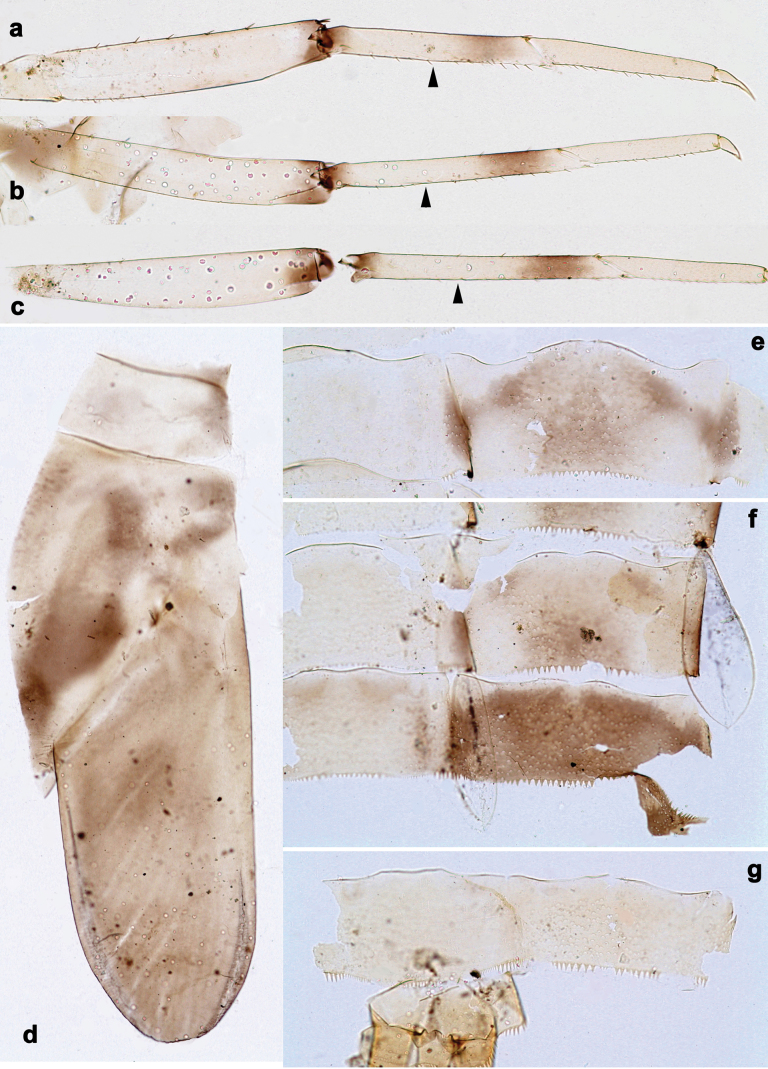
*Arcobaetissripadai* sp. nov., larval exuviae (with same magnification): **a–c** fore, middle and hind legs (triangles show points where patella-tibial suture crosses inner margin) **d** half of pronotum and mesonotum **e** sternum and tergum V **f** sterna and terga VII–VIII **g** sterna and terga IX–X.

**Figure 14.**
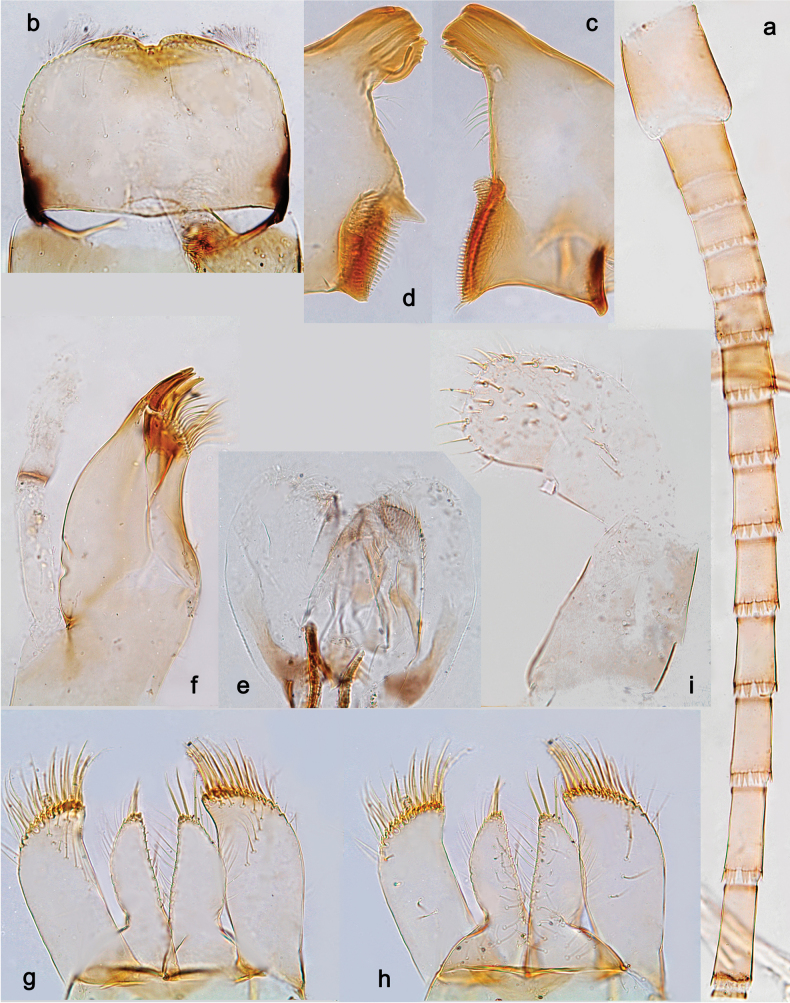
*Arcobaetissripadai* sp. nov., larval exuviae (with same magnification) **a** antenna **b** labrum (dorsal side) **c** right mandible **d** left mandible **e** hypopharynx and superlinguae **f** maxilla **g** labium (dorsal focus) **h** labium (ventral focus) **i** labial palp.

**Figure 15.**
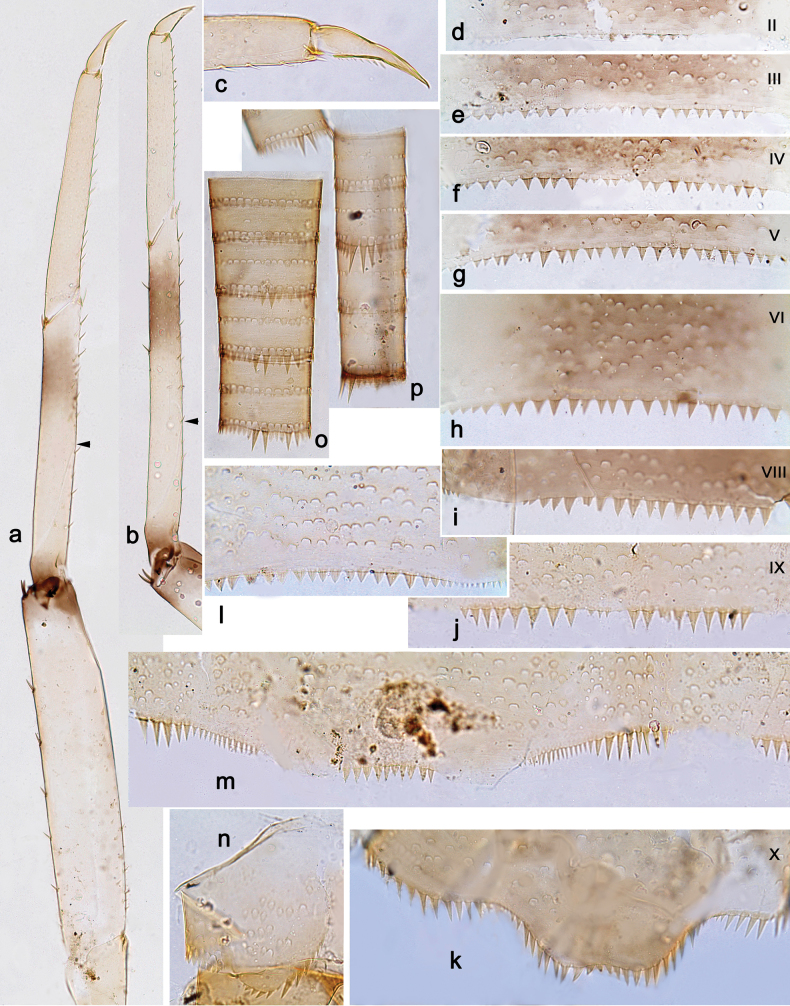
*Arcobaetissripadai* sp. nov., larval exuviae **a, b** fore and middle legs (triangles show points where patella-tibial suture crosses inner margin **c** claw **d–k** posterior margins of abdominal terga II–VI and VIII–X **l–m** posterior margin of abdominal sterna VIII and IX of male larva **n** paraproct. **o–p** fragments of cerci (lateral view).

**Figure 16.**
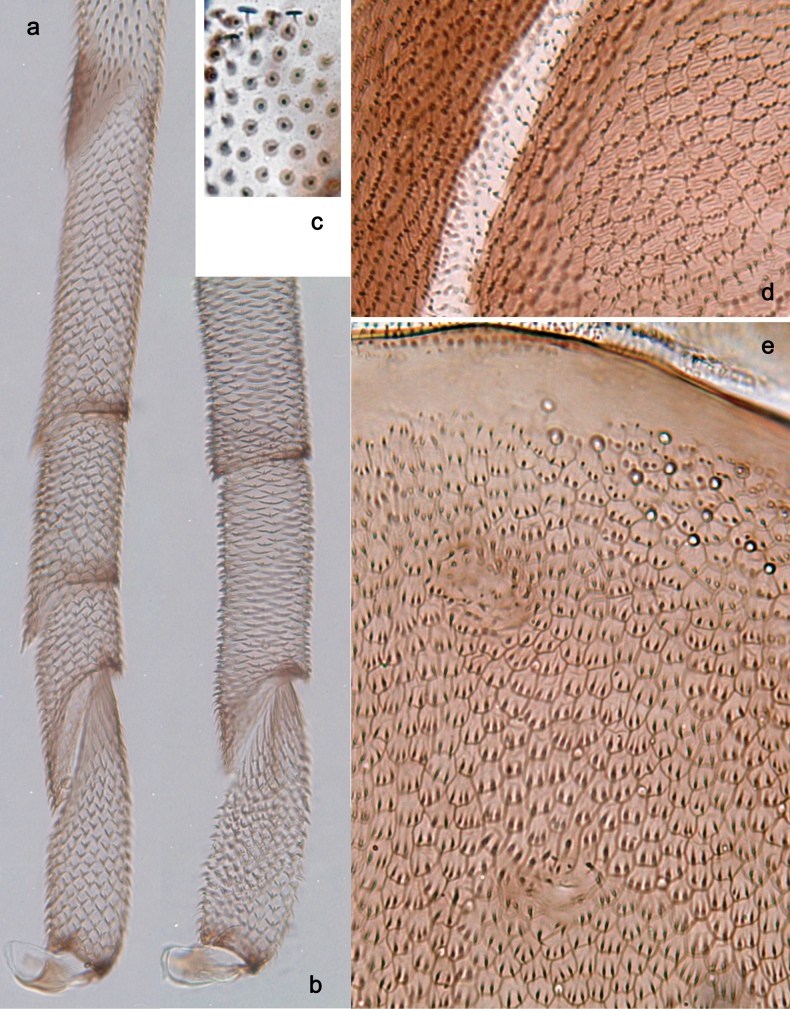
*Arcobaetissripadai* sp. nov., subimaginal exuviae **a** hind tarsus **b** apex of fore tarsus **c** wing membrane **d** fragment of mesonotum **e** fragment of abdominal tergum VII with two right submedian sigilla.

**Figure 17.**
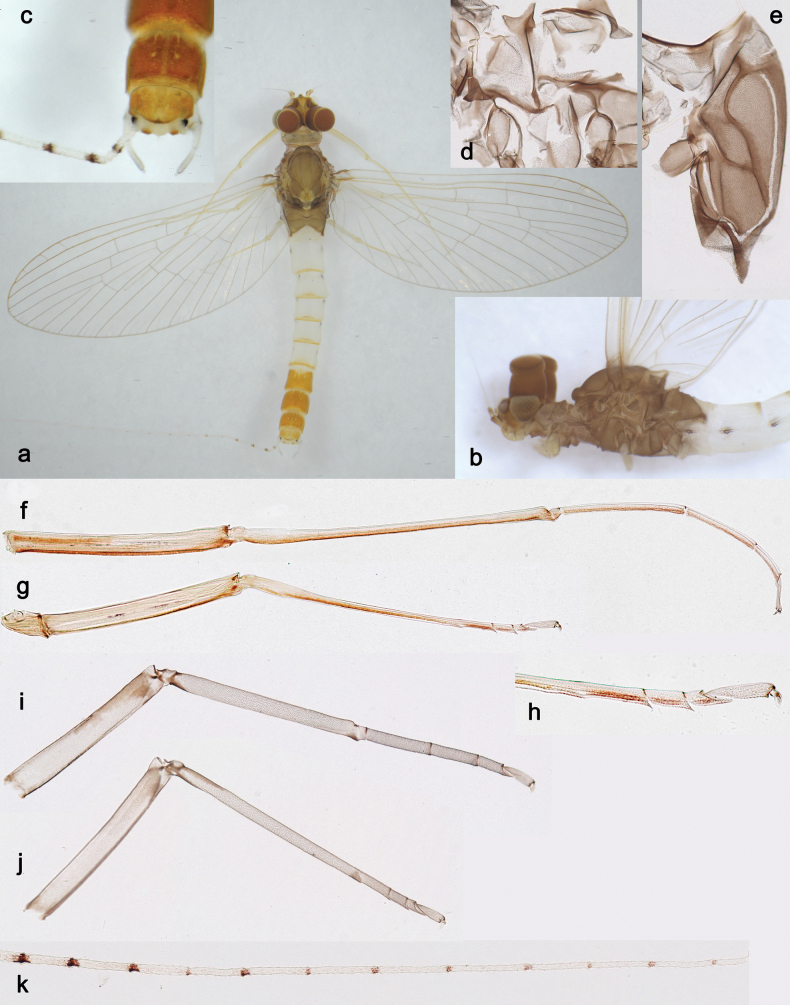
*Arcobaetissripadai* sp. nov. **a** male imago **b** head and thorax **c** apex of abdomen **d** subimaginal exuviae of meso- and metapleura **e** subimaginal exuviae of half of mesonotum **f, g** fore and middle legs **h** tarsus of middle leg **I, j** subimaginal exuviae of fore and middle legs **k** cercus.

**Figure 18.**
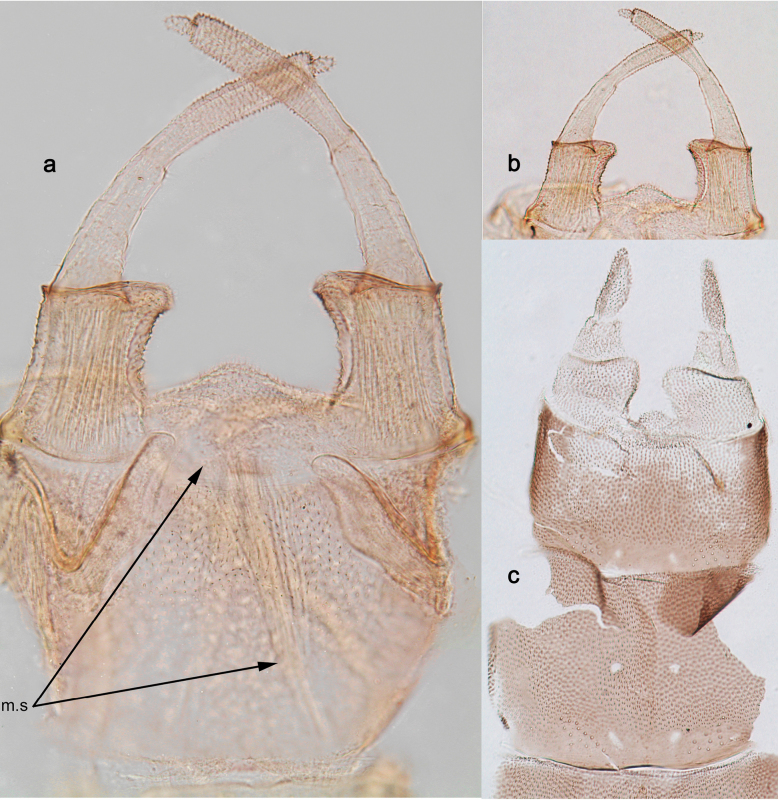
*Arcobaetissripadai* sp. nov. **a** genitalia of male imago **b, c** imaginal genitalia and their subimaginal exuviae with the same magnification. Abbreviation: **m.s** asymmetric sterno-styligeral muscle (arrows show its base and apex)

**Figure 19.**
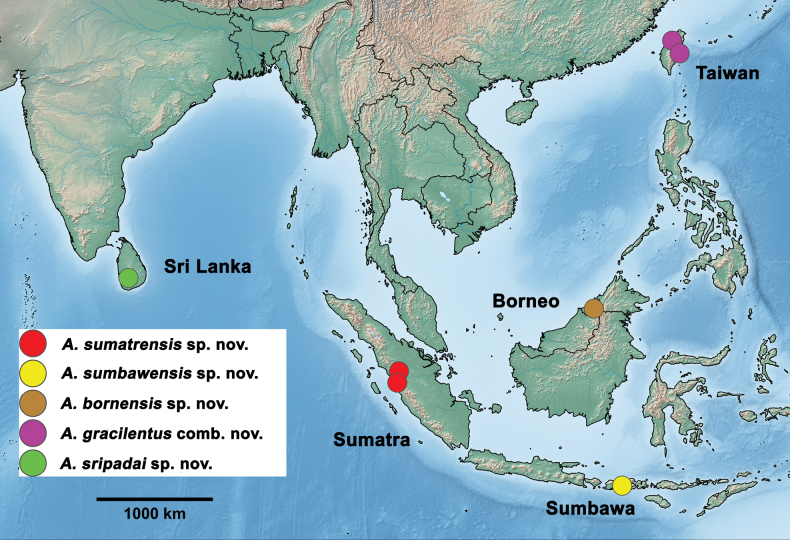
*Arcobaetis* gen. nov., distribution.

Figs 13 , 14 , 15 , 16 , 17 , 18 , 19

##### Type material.

***Holotype*.** ♂ imago reared from larva, with its larval and subimaginal exuviae {specimen [XVIII] (5)2020}, Sri Lanka, foot of Sri Pada (Adam’s Peak), Delhausie, river Seetha gangula; 6°50′3.48″N, 80°34′3.36″E; 7.II.2020; leg. N. Kluge & L. Sheyko; SPbU.

##### Diagnosis.

**Larva** (Table 1). The following combination of characters distinguish *A.sripadai* sp. nov. from other species of *Arcobaetis* gen. nov.: A) distal margins of segments in middle part of flagellum without enlarged spines (Fig. 14a); B) labial palp segment III sub-quadrangular, at base approx. as wide as distal margin of segment II (Fig. 14i); C) hind protoptera absent; D) claw with single row of denticles, most distalmost denticles larger, basal denticles small to minute (Fig. 15c); E) tergalii present on abdominal segments II–VII; F) posterior margin of tergum IV with triangular spines, slightly wider than long (Fig. 15f).

##### Descriptions.

**Larva** (Figs 13–18).

***Cuticular colouration*** (Fig. 13a–g). Head, thoracic terga and pleura with diffusive brown, ochre, and colourless areas (Fig. 13d); thoracic sterna colourless. Legs pale ochre with brown apex of femur, base of tibia and band in distal part of tibia (Fig. 13a–c). Abdominal terga I–VII with nearly uniform colour pattern consisting of diffusive ochre and pale brown areas (Fig. 13e–f); tergum VIII nearly uniformly darker brown, with paler ochre anterior margin and anterolateral angles (Fig. 13f); terga IX–X uniformly pale ochre (Fig. 13g); all sterna I–IX and paraprocts pale ochre. Caudalii ochre.

***Hypodermal colouration.*** Unknown.

***Antenna*** (Fig. 14a). Spines at distal margins of flagellum segments not enlarged.

***Labrum*** (Fig. 14b). Length 0.7× maximum width. Dorsally with submedian setae and few long setae, not forming distinct submarginal arcs.

***Right mandible*** (Fig. 14c). Number of denticles of incisor unclear (worn); kinetodontium with three denticles.

***Left mandible*** (Fig. 14d). Number of denticles of incisor unclear (worn); kinetodontium with three denticles.

***Hypopharynx and superlinguae*** (Fig. 14e). Lingua shorter than superlinguae, longer than broad. Superlinguae distally almost straight; lateral margins rounded; fine, long, simple setae along distal margin.

***Maxilla*** (Fig. 14f). Galea-lacinia ventrally with two simple setae just proximad of canines. Medially with one spine-like seta and four simple setae. Maxillary palp approx. 1.1× as long as galea-lacinia; palp segment II slightly shorter than segment I.

***Labium*** (Fig. 14g, h). Inner margin of glossa with 14 or 15 spine-like setae, increasing in length distally; apex with three long and robust setae; outer margin with ca. ten spine-like setae; paraglossa ventrally with ca. four medium, simple setae in anteromedial area; dorsally with arc of ca. ten long, spine-like setae in distal area. Labial palp with segment I 0.8× length of segments II and III combined. Labial palp segment II dorsally with row of three or four spine-like setae. Segment III sub-quadrangular; at base approx. as wide as distal margin of segment II; ventral surface with short, spine-like, simple setae and short, fine, simple setae.

***Hind protoptera*** absent.

***Legs*** (Figs 15a–c). Long and slender; middle and hind legs slenderer than fore legs; hind leg slightly longer than others; on fore leg, tibia and tarsus of subequal length, with patella-tibial suture on proximal ¹/2 of tibia; on middle and hind legs, tibia much longer than tarsus, with patella-tibial suture on proximal ¹/3 of tibia. Segments ratio of fore leg 1.4:1.0:1.0:0.2, middle leg 1.3:1.0:0.7:0.2, hind leg 1.2:1.0:0.7:0.2. ***Femur*.** Length 4.6× maximum width. Outer margin with row of 5–7 short, spine-like setae, larger on fore leg and smaller on middle and hind legs; two apical setae larger, equal on all legs (Fig. 15a). Ventral side of femur with smaller pointed spine-like setae, larger and denser on fore legs, smaller and fewer on middle and hind legs. ***Tibia.*** Pointed, feathered, spine-like setae irregularly located on inner side of fore tibia (Fig. 15a) and on all sides of middle and hind tibiae (Fig. 15b); one preapical seta on outer side of each tibia. ***Tarsus.*** Ventral margin with row of short, curved, feathered, spine-like setae. ***Claw*** with one row of ca. ten denticles increasing from basal to distal ones and directed distally (Fig. 15c).

***Abdominal terga*** (Fig. 15d–k). Posterior margin of terga: I smooth, without spines; II–X with triangular spines, increasing in length toward X; slightly longer than wide on tergum IV; row of spines on tergum IX interrupted behind bases of submedian setae (Fig. 15j). Posterior margin of tergum X with median concavity with smaller spines in it (Fig. 15k).

***Abdominal sterna*** (Fig. 15l–m). Posterior margins of sterna: I–V smooth, without spines; VI–VIII with triangular spines (Fig. 15l). On sternum IX of male row of narrow pointed spines between protogonostyli, smaller and narrower spines laterad of protogonostyli and larger pointed spines laterad of them (Fig. 15m).

***Paraproct*** (Fig. 15n). Posterior margin with ca. 16 stout spines. Surface scattered with scales.

***Caudalii*** (Fig. 15o–p). Cerci and paracercus with small, elongate spines on posterior margin of each segment; on outer side of cercus several spines on each 2^nd^ segment greatly enlarged and pointed; no such enlarged spines on paracercus.

***Tergalii*** (Fig. 13f). Present on abdominal segments II–VII, subequal.

**Subimago** (Figs 16a–e, 17d, e, I, j).

***Cuticular colouration.*** Head and prothorax mostly brown. Mesonotum brown, medioparapsidal suture contrastingly colourless (Figs 16d, 17e). Meso- and metathoracic pleura and sterna brown with colourless areas (Fig. 17d). Wing membrane colourless with microtrichial circles ring-like, brown (Fig. 16c). Legs mostly colourless with microtricha and microlepides dark brown, with brown markings on femur, at base and apex of tibia and margins of tarsomeres (Figs 16a, b, 17i, j). Abdominal terga I-X and sterna I-VI uniformly brown, with all sigilla of the same colour as background; sterna VII-VIII with submedian sigilla paler than background, sternum IX of male with distal part and gonostyli colourless (Fig. Fig. 16e). Cerci colourless with setae dark brown.

***Texture*.** Mesonotum with cross-striated polygonal areas bordered with microtrichia (Fig. 16d). Abdominal terga and sterna with outlined polygonal areas bearing two or more microtrichia each; sigilla diminished (Fig. 16e). On fore leg of male, 1^st^–4^th^ tarsomeres covered with blunt microlepides, 5^th^ tarsomere covered with pointed microlepides; on middle and hind legs, all tarsomeres co­vered with pointed microlepides (Fig. 16a, b).

**Male imago** (Fig. 17a–c, f–h, k). Head ochre with reddish. Antennae with scape and pedicel ochre with reddish-brown apices. Turbinate eyes narrow, cylindrical, red. Thorax ochre-brownish. Fore wings with membrane colourless, proximal portions of C and Sc+RA reddish-brown, other veins ochre-brownish. Pterostigma with ca. three crossveins. Hind wings absent. Legs of all pairs ochre with inner side reddish (Fig. 17f–h). Middle and hind tarsi with two apical spines (on 1^st^ +2^nd^, and 3^rd^ segments). Abdominal segments I–VI white with yellow stripe on posterior margin of each tergum and blackish spot on each stigma; terga VII-X yellow-ochre. Cerci whitish, with brown marking at each joining (Fig. 17k). Gonostyli whitish.

***Genitalia*** (Fig. 18a–c). Unistyligers widely separated, with shallow, not sclerotised, conic projection between them. Each unistyliger cylindrical, distally widened and projected medially. First segment of gonostylus narrowing toward apex, poorly separated from 2^nd^ segment. 3^rd^ (apical) segment extraordinary small, much narrower than apex of 2^nd^ segment. Gonovectes sharply bent. Sterno-styligeral muscle distinctly developed, but attached far from anterior margin of sternum; in single examined specimen sharply asymmetric.

***Dimension.*** Fore wing length (and body length) 3.5 mm.

##### Etymology.

Specific epithet refers to the type locality at the foot of the Sri Pada (Adam’s Peak).

##### Distribution.

Sri Lanka (Fig. 19)

#### ﻿ Arcobaetis gracilentus comb. nov.

Taxon classificationAnimaliaEphemeropteraBaetidae

FA4784A8-7F05-5F50-83C7-7898CD00379D

Nigrobaetis gracilentus : Kang et al. 1994: fig. 7A–L.

##### Diagnosis.

**Larva.** Following combination of characters distinguish *Arcobaetisgracilentus* comb. nov. from other species of *Arcobaetis* gen. nov.: A) distal margins of segments in middle part of flagellum without long spines; B) labial palp segment III sub-quadrangular, at base narrower than distal margin of segment II (Kang et al. 1994: fig. 7E); C) hind protoptera present (Kang et al. 1994: fig. 7G); D) tergalii present on abdominal segments I–VII; E) posterior margin of tergum VI with triangular spines, longer than wide (Kang et al. 1994: fig. 7K).

##### Distribution.

Taiwan.

### ﻿Key to the species of *Arcobaetis* gen. nov. (larvae)

**Table:** 

1	Tergalii present on abdominal segments II-VII	**2**
–	Tergalii present on abdominal segments I-VII	**3**
2	Posterior margin of each 2^nd^ segment of cerci with several enlarged, sharply pointed spines on lateral side (Fig. 15o, p). Spines on posterior margins of abdominal terga long and narrow (Fig. 15f–k)	***A.sripadai* sp. nov.**
–	Posterior margin of each 2^nd^ segment of cerci with two slightly enlarged, pointed spines on lateral side (Fig. 9f). Spines on posterior margins of abdominal terga shorter and wider (Fig. 8i)	***A.sumatrensis* sp. nov.**
3	Hind protoptera present	***A.gracilentus* comb. nov.**
–	Hind protoptera absent	**4**
4	Flagellum in middle part with enlarged spines at distal margins of segments (Fig. 10f); labial palp segment III sub-quadrangular, at base narrower than distal margin of segment II (Fig. 9h)	***A.bornensis* sp. nov.**
–	Flagellum in middle part without enlarged spines at distal margins of segments; labial palp segment III sub-rectangular, at base as wide as distal margin of segment II (Fig. 2h)	***A.sumbawensis* sp. nov.**

### ﻿Genetics

The two COI sequences obtained from specimens of *A.sumbawensis* sp. nov. from the same location have a genetic distance of 0% (K2P), as it is expected in such a case.

### ﻿Distribution

## ﻿Discussion

### ﻿Relationship, characters, and affinities of *Arcobaetis* gen. nov.

The new genus *Arcobaetis* gen. nov. obviously belongs to the family Baetidae based on the turban eyes of the male imago (Fig. 17b), the fore wing with intercalary veins (Fig. 17a), and a series of larval characters, e.g., Y-shaped frontal suture ventral of lateral ocelli (Fig. 4e), labrum with distinctly expressed medial incision (Fig. 2a), kinetodontium fused with mandible and with incisor (Fig. 2b, d), left prostheca stout and stick-like, apically denticulate (Fig. 2d, e), femur with apical anterior outer projection curved toward inner side of femur (Fig. 3a) (Kluge 2004; Kluge and Novikova 2011).

Based on the rank-free system of Kluge (Kluge 2004; Kluge and Novikova 2011), *Arcobaetis* gen. nov. belongs to the Anteropatellata because the patella-tibial suture is present on all legs of the larva, including fore legs (Fig. 4a–c); to Baetovectata because of the forewings with double intercalaries (Fig. 17a), the characteristic structure of the gonovectes (Fig. 18a), and the 2^nd^ segment of the subimaginal gonostylus developing under larval cuticle bent caudally or medially, but not laterally (Fig. 4d); and to Baetungulata or Baetinae (sensu Kazlauskas 1972) because of the claws with one row of denticles on inner-anterior side and a maxillary palp with two segments (Figs 2g, 5a) (Kluge and Novikova 2011). Finally, the new genus is part of the “non-Baetofemorata” or the “non-*Baetis* complex” sensu Waltz and McCafferty (1997), because larval legs have no femoral patch and male subimago has tarsomeres of middle and hind legs and 5^th^ tarsomere of fore leg covered with pointed microlepids (Figs 3a, 16a, b) (Kluge and Novikova 2011).

The genus *Arcobaetis* gen. nov. is closely related to *Nigrobaetis* s.l. (incl. subgenera or genera *Nigrobaetis* s.str., *Takobia* Novikova & Kluge, 1987, *Alainites* Waltz & McCafferty, 1994, *Margobaetis* Kang & Yang, 1994 and *Diphetor* Waltz & McCafferty, 1987), sharing several larval characters, e.g., frons with carina-like ele­vation; labial palp segment II not greatly projected; abdominal terga with scales in trapezoidal nests with corner opercula; subimaginal gonostyli developing under cuticle of last instar male larva folded in “*Nigrobaetis*-type”. *Arcobaetis* gen. nov. is distinguished from *Nigrobaetis* s.l. by paraglossae with a dorsal arc of long, spine-like setae in distal area, and both mandibles with long, slightly feathered setae between prostheca and mola. *Nigrobaetis* s.l. has no arc of setae on paraglossae and usually has short denticles between prostheca and mola of left mandible or a smooth margin (Müller-Liebenau 1984; Kaltenbach and Gattolliat 2023); only in rare cases, there is a medium tuft or short row of setae close to prostheca of left mandible (*N.colonus* Gattolliat, 2004; *N.cryptus* Gattolliat, 2004; *N.terminus* Chang & Yang, 1994) (Kang et al. 1994; Gattolliat 2004). Further, *Arcobaetis* gen. nov. has very slender legs with femora length 4–6× maximum width, outer margin of femora with a row of short, spine-like setae, and claws with distal denticles larger and directed distad and proximal denticles minute. *Nigrobaetis* s.l. usually has less slender legs, outer margin of femora with a row of long, spine-like setae (apart from some *Alainites* and *Takobia* that also have short, spine-like setae at outer margin of femur), and claws with denticles gradually decreasing in size toward base or exceptionally without denticles (Müller-Liebenau 1984; Sroka et al. 2021; Yanai et al. 2022; Phlai-ngam et al. 2022b; Kaltenbach and Gattolliat 2023). Additionally, the larvae of *Alainites* are distinguished by a paraproct with an elongated prolongation, and the prostheca of the right mandible is bifid, reduced to two bristle-like feathered appendages (Yanai et al. 2022), both cha­racters are absent in *Arcobaetis* gen. nov.; the larvae of *Takobia* have a paraproct with a short, bent prolongation and a right prostheca reduced to split bristles (Sroka et al. 2021), whereas these characters are absent in *Arcobaetis* gen. nov.

The larvae of *Procerobaetis* Kaltenbach & Gattolliat, 2020, which is another related genus, have remarkable long, pointed tergalii (especially tergalii I and II) (Kaltenbach et al. 2020b), whereas *Arcobaetis* gen. nov. has the usual more or less ovoid tergalii.

The male imago of *Arcobaetissripadai* sp. nov. has an extraordinarily small 3^rd^ (apical) segment of gonostylus, much narrower than apex of 2^nd^ segment. This is not the case in *Nigrobaetis* s.l., where the 3^rd^ segment of gonostylus of male imagoes has the usual size, being approx. as wide as the 2^nd^ segment (Müller-Liebenau 1969).

### ﻿Distribution

**Table 2.** GPS coordinates of locations of *Arcobaetis* gen. nov.

Species	Location	Coordinates
*A.sumbawensis* sp. nov	Indonesia: Sumbawa	08°37'42"S, 117°15'27"E
*A.sumatrensis* sp. nov.	Indonesia: Sumatra	00°06'26"S, 100°40'22"E
		00°56'44"S, 100°32’ 44"E
*A.bornensis* sp. nov.	Brunei	04°32'55"N, 115°09'27"E
*A.sripadai* sp. nov.	Sri Lanka	06°50'03"N, 80°34'03"E
*A.gracilentus* comb. nov.	Taiwan	24°28'19"N, 120°58'37"E
		23°32'13"N, 121°31'42"E

*Arcobaetis* gen. nov. has a very wide distribution across South Asia, so far including Indonesia (Sumatra, Sumbawa), Brunei (Borneo), Sri Lanka, and Taiwan (Fig. 19). Taking into account the generally high diversity in this vast region, and the rather limited collection activities in the past, with many still unexplored regions, we have to expect more species and an even larger distribution, including most of continental South Asia, and probably also the Philippines. No species outside the Oriental realm is in line with the diagnoses of *Arcobaetis* gen. nov. and could be assigned to this genus.

## Supplementary Material

XML Treatment for Arcobaetis

XML Treatment for Arcobaetis sumbawensis

XML Treatment for Arcobaetis sumatrensis

XML Treatment for Arcobaetis bornensis

XML Treatment for Arcobaetis sripadai

XML Treatment for Arcobaetis gracilentus

## ﻿Additional information

### Author ORCIDs

Thomas Kaltenbach https://orcid.org/0000-0001-8052-0388

Nikita J. Kluge https://orcid.org/0000-0001-9741-7790

Jean-Luc Gattolliat https://orcid.org/0000-0001-5873-5083

### Data availability

All of the data that support the findings of this study are available in the main text or Supplementary Information.
